# Identification of a gene conferring broad-spectrum orthotospovirus resistance in Solanaceae

**DOI:** 10.1126/sciadv.adw4333

**Published:** 2025-06-18

**Authors:** Yong Liu, Jie Wang, Chongkun Zuo, Weishu Fan, Cheng Yuan, Jianmin Zeng, Haiqin Yu, Zhijun Tong, Xueyi Sui, Yi Xu, Min Zhu, Xiaorong Tao, Jiongjiong Chen, Hanhui Kuang, Polina Yu. Novikova, Changjun Huang

**Affiliations:** ^1^ Key Laboratory of Tobacco Biotechnological Breeding, Yunnan Daguan Laboratory, China Tobacco Breeding Technology Innovation Center, Yunnan Academy of Tobacco Agricultural Sciences, Kunming 650021, China.; ^2^ Department of Chromosome Biology, Max Planck Institute for Plant Breeding Research, Cologne 50829, Germany.; ^3^ The Key Laboratory of Plant Immunity, Department of Plant Pathology, Nanjing Agricultural University, Nanjing 210095, China.; ^4^ Institute of Genetics and Developmental Biology, Chinese Academy of Sciences, Beijing 100101, China.; ^5^ National Key Laboratory for Germplasm Innovation and Utilization of Horticultural Crops, Hubei Hongshan Laboratory, College of Horticulture and Forestry Sciences, Huazhong Agricultural University, Wuhan 430070, China.

## Abstract

Linkage drag can hinder the integration of resistance genes from wild crop relatives into breeding programs. We used a chromosome-scale *Nicotiana alata* genome assembly and a segregating population exceeding 160,000 plants to dissect the complex genetic architecture and overcome the tight linkage between resistance and deleterious loci to produce plants free from linkage drag. We cloned *N. alata RTSW*, encoding an immune receptor that confers broad-spectrum resistance to orthotospoviruses through the interaction of its carboxyl-terminal domain with an orthotospovirus-encoded protein. Notably, despite recognizing the same avirulence factor, *RTSW* genes from *N. alata* and *Sw-5b* from *Solanum peruvianum* have evolved independently of adjacent nonorthologous ancestral loci. Our work illustrates the potential of wild relative genomes as resources from which to precisely introduce disease resistance into cultivated crops.

## Teaser

Wild tobacco genome unravels decades-old breeding barrier, enabling broad-spectrum virus-resistant crop development.

## INTRODUCTION

Spotted wilt disease—caused by orthotospoviruses of the order Bunyavirales, family *Tospoviridae*, genus *Orthotospovirus*—seriously threatens crops and causes substantial economic losses (*1*, *2*). On the basis of the sequence of the virus nucleoprotein N, orthotospoviruses are classified into two main phylogenetic groups corresponding to their primary geographical origins: Eurasian and American. Tomato spotted wilt orthotospovirus (TSWV), in the American group, is the second most destructive plant virus worldwide, infecting more than 1000 plant species including valuable vegetables, legumes, and ornamentals, along with weeds (*3*–*5*). Orthotospoviruses are transmitted through thrips in the genus *Frankliniella*, which are nearly impossible to control owing to their small size, rapid development, high reproduction rate, and resistance to insecticides (*6*). Breeding resistant crop cultivars is therefore considered the most effective approach (*7*, *8*).

Two genes conferring resistance to TSWV have been cloned: *Spotted wilt resistance 5b* (*Sw-5b*) from wild tomato (*Solanum peruvianum*) and *resistance against Tomato spotted wilt orthotospovirus* (*Tsw*) from habanero pepper (*Capsicum chinense*) (*9*, *10*). Sw-5b and Tsw are nucleotide-binding leucine-rich repeat (NB-LRR or NLR) immune receptors with an N-terminal coiled-coil (CC) domain (CNL) that induce resistance upon recognition of the nonstructural protein m (NSm) and nonstructural protein s (NSs) produced by TSWV (*11*–*13*). *Tsw* provides specific resistance against TSWV, while *Sw-5b* confers broader resistance against American-type orthotospoviruses; neither is effective against Eurasian orthotospoviruses (*9*, *14*, *15*). The rapid spread of Eurasian orthotospoviruses, together with reports of American-type orthotospoviruses breaking the resistance conferred by *Sw-5b* and *Tsw*, highlights the urgent need to develop new resistance genes (*2*, *7*).

Wild crop relatives are natural reservoirs of resistance genes (*16*). The annual contribution of resistance traits transferred from wild relatives to domesticated crops was estimated to be around US$186.3 billion globally in 2020 (*17*). However, the introgression of desired loci is time consuming, high risk, and labor intensive, owing to interspecies barriers and linkage drag (*18*). Specifically, linked deleterious genes introduced alongside the desired gene decrease the agronomic fitness of progeny between elite cultivars and wild relatives, making new cultivars with improved traits “nearly inaccessible” to breeders (*17*).

Jasmine tobacco (*Nicotiana alata*), a wild diploid species, is the only known natural source of resistance to TSWV conferred by the *RTSW* (also known as *RSTV-al*) locus within the *Nicotiana* genus (*19*, *20*). *RTSW* was subsequently introgressed into allotetraploid cultivated tobacco (*Nicotiana tabacum*) using *Nicotiana otophora* as a bridging parent, leading to the development of the breeding line “Polalta” in the 1980s (*21*). However, the Polalta line exhibits strong morphological deformities such as thickened and ribbon-shaped leaves, irregular venation, and dwarfing (*22*–*25*), suggesting a close linkage between *RTSW* and *DEFORMITY* (*DEF*) loci underlying these morphological aberrations. Despite the development of several *RTSW*-related markers and numerous breeding strategies used over the past four decades, the production of TSWV-resistant tobacco cultivars free of morphological defects has not yet been achieved (*22*, *23*, *26*).

In this study, we assembled the *N. alata* genome for a reference-guided approach to examining the intricate genetic relationship between the *RTSW* and *DEF* loci and overcoming their genetic linkage. We annotated the *N. alata* genome assembly, with a focus on NLR gene expansion relative to other Solanaceae species. From a set of more than 160,000 segregating individuals derived from a seventh backcross (BC) between Polalta and *N. tabacum*, we isolated *RTSW* orthotospovirus-resistant plants with no morphological defects, culminating in the development of a resistant variety for breeding programs. We also identified the resistance gene *RTSW* within an expanded NLR gene cluster in *N. alata* responsible for TSWV resistance. We assessed whether *RTSW* confers broad resistance against both American and Eurasian orthotospoviruses and explored its potential to provide resistance to other Solanaceae crops. We also used syntenic and phylogenetic signals to compare the evolutionary relationship between *RTSW* from *N. alata* and *Sw-5b* from *S. peruvianum*, which provide resistance to TSWV through recognition of the same avirulence (Avr) protein. The identification of *RTSW* will facilitate the development of RTSW-resistant cultivars in Solanaceae crops and beyond.

## RESULTS

### Assembly of the *N. alata* genome and phylogenetic analysis of NLR genes

**Fig. 1.**
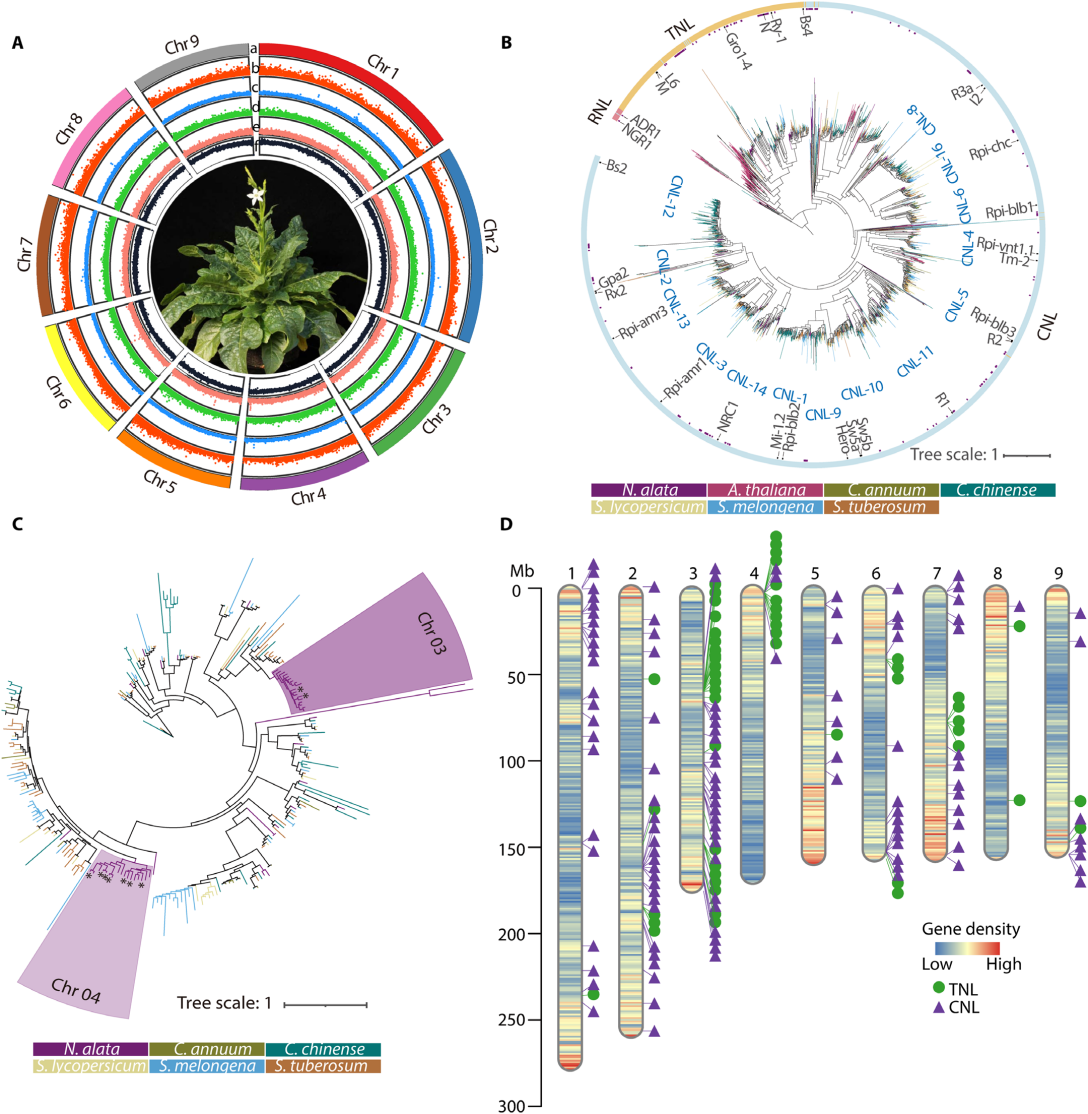
Architecture of the *N. alata* genome and annotation of its NLR genes. (**A**) Circos plot depicting genome features across the nine *N. alata* chromosomes. (a) *N. alata* chromosomes; (b) gene density (protein-coding and noncoding); (c to e) distribution of repeat elements: *Gypsy* (green, c), *Copia* (yellow, d), and tandem repeats (blue, e); and (f) guanine and cytosine (GC) content (light brown). Sliding window size = 200 kb. The central region shows an *N. alata* plant. Chr1, chromosome 1. (**B**) Phylogenetic analysis of NLR family members from *N. alata* and other Solanaceae species. The maximum-likelihood tree, including 2383 annotated NLR proteins and 26 reference NLR proteins (marked within their respective clades), was constructed with IQ-TREE. The outer circle highlights the major clades of NLRs: the Toll/interleukin-1 receptor/resistance (TIR) domain type NLR (TNL), CNL, and resistance to powdery mildew 8 domain type NLR (RNL) subfamilies. The NLRs from *N. alata* are highlighted by purple bars inside the outer circle. The phylogenetic tree was rooted in *Arabidopsis thaliana* NLR proteins and is color coded to indicate different species. The tree scale is indicated, with branch lengths proportional to the number of amino acid substitutions per site. (**C**) Phylogenetic relationships of TNLs identified from the *N. alata* genome sequence and selected Solanaceae species. Two specific TNL subgroup expansions in *N. alata* are highlighted with dark and light purple backgrounds. Asterisks indicate genes not located on chromosome 3 or 4. (**D**) Distribution of TNL and CNL genes across the *N. alata* genome. CNL and TNL genes are represented as purple triangles and green dots, respectively, across the nine *N. alata* pseudochromosomes. The ideograms display gene density (bin size: 1 Mb) using a gradient from blue (low) to red (high).

An estimation of the *N. alata* genome size revealed a relatively large heterozygous genome of 1.77 to 1.87 gilbert (Gb) with a heterozygosity of 0.68%, attributed to self-incompatibility (fig. S1, A and B) (*27*). To produce a chromosome-level assembly, we obtained Illumina paired-end (366-fold coverage), mate-pair (486-fold coverage), 10x Genomics (116-fold coverage), and high-throughput chromosome conformation Capture (Hi-C) (108-fold coverage) sequencing reads (data S1). We assembled the genome using DeNovoMAGIC3 and scaffolded the contigs with three-dimensional (3D) DNA, resulting in a final assembly of 1.761 Gb, with 97.1% (1.71 Gb) anchored to nine pseudomolecules (Fig. 1A, fig. S1C, and data S2 to S4), corresponding to the haploid chromosome number of *N. alata*. The Benchmarking Universal Single-Copy Orthologs (BUSCO) score was 94.6% for all complete single-copy and duplicated orthologs, supporting the completeness of this assembly (data S5). Repeats accounted for 81.4% of the *N. alata* genome (data S6). We also identified 33,177 protein-coding genes, 90.8% of which were functionally annotated (data S7 to S10). The protein-coding and noncoding genes are unevenly distributed across the genome, with protein-coding genes increasing in frequency toward the ends of the chromosomes (Fig. 1A).

To explore the evolution and molecular basis of TSWV resistance, we annotated all NLR genes encoding immune receptors that recognize pathogen effectors (also referred to as Avr factors) and trigger immune response (*28*–*30*). Our previous identification of the TSWV NSm protein as the Avr factor for the *RTSW* locus (*20*) suggested that the *RTSW* locus in *N. alata* is an NLR gene, in accordance with Flor’s gene-for-gene hypothesis (*31*). Using an optimized domain-based search and manual curation, we identified 337 NLR genes in the *N. alata* genome, including 56 Toll/interleukin-1 receptor/resistance (TIR)–NB-LRR (TNL) genes, 117 CNL genes, 2 resistance to powdery mildew 8–NB-LRR (RNL) genes, and 162 partial/truncated NLR sequences lacking one or more of the canonical NLR-family domains (data S11). We constructed a phylogenetic tree from protein alignments of selected NLRs from five representative Solanaceae species [pepper (*Capsicum annuum*), habanero pepper (*C. chinense*), tomato (*Solanum lycopersicum*), eggplant (*Solanum melongena*), and potato (*Solanum tuberosum*)] and included 166 *Arabidopsis thaliana* NLRs with intact domains as an outgroup (Fig. 1B). The TNL, RNL, and CNL clades were deeply separated, reflecting the ancient divergence of these subclasses. RNL was further separated into two clades, while the CNL clade was further divided into 14 subgroups (Fig. 1B). We detected species-specific expansion and contraction of the CNL family, which is common in Solanaceae and consistent with previous reports (*32*). Notably, in *N. alata*, apart from CNL expansion, certain subgroups of TNLs were expanded, especially on chromosomes 3 and 4, where these expanded groups form clusters (Fig. 1, C and D).

### Overcoming the tight linkage of *RTSW* and *DEF*

**Fig. 2.**
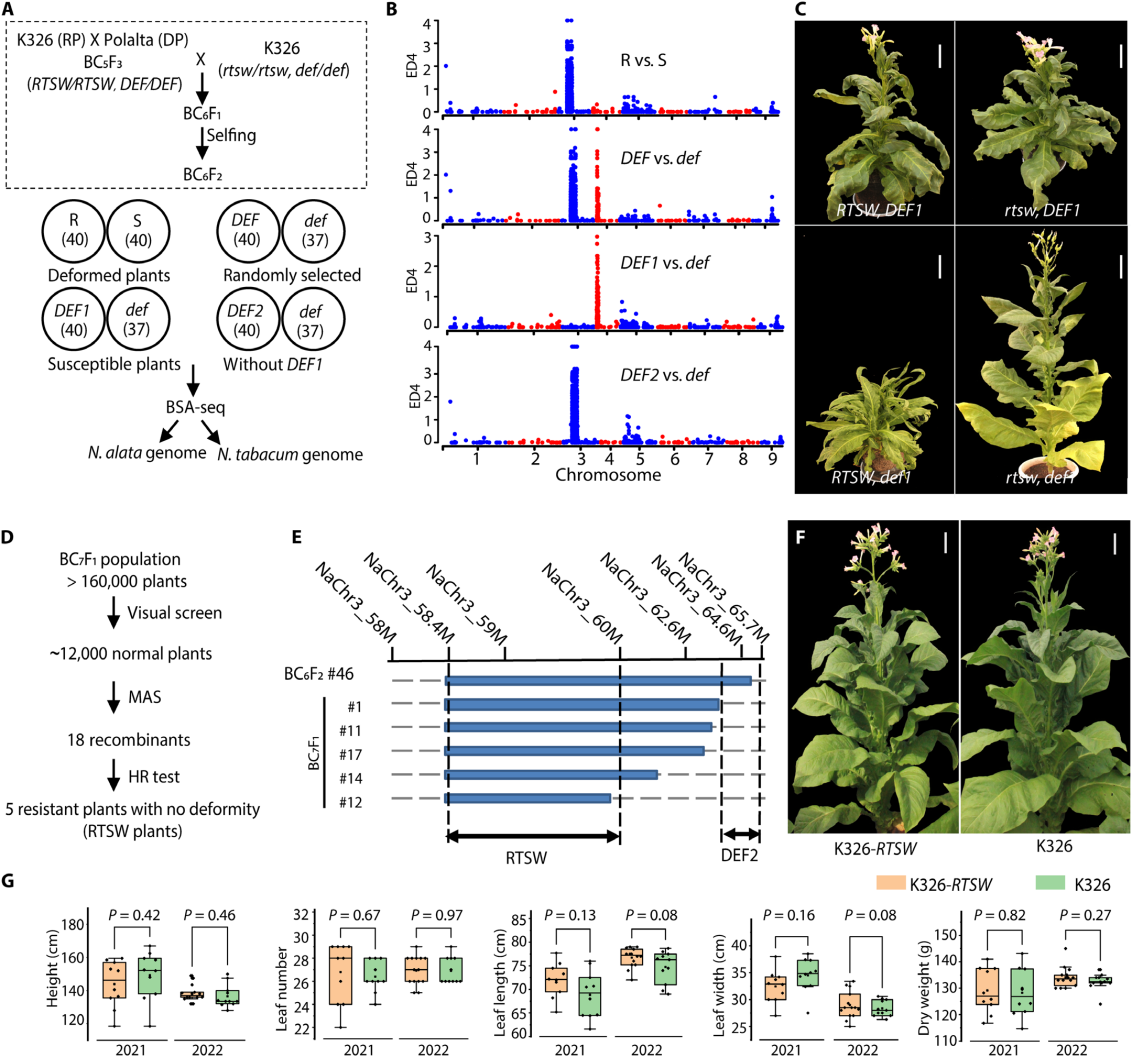
Genetic analysis of resistance and deformity traits and acquisition of resistant plants with no deformity. (**A**) Diagram of the strategy for BSA-seq analysis of TSWV resistance and plant deformity. A Polalta × *N. tabacum* K326 (K326, recurrent parent) BC_6_F_2_ population was used for phenotyping (top). Four pooled groups [resistant (R), susceptible (S), deformed (DEF), and normal (def); DEF1 and DEF2] were analyzed (pool sizes in parentheses; bottom). RP, recurrent parent; DP, donor parent. (**B**) Euclidean distance (ED) analysis of single-nucleotide polymorphism (SNP) frequency differences between phenotypic groups (as described above) along the *N. alata* genome. (**C**) Representative photographs of the four different genotype groups: plants with both *RTSW-DEF2* and *DEF1* (*RTSW* and *DEF1*), plants with only *DEF1* (*rtsw* and *DEF1*), plants with only *RTSW-DEF2* (*RTSW* and *def1*), and plants without either *RTSW-DEF2* or *DEF1* (*rtsw* and *def1*). Scale bars, 10 cm. (**D**) Strategy to isolate resistant plants with no deformity (*RTSW*, *def1*, and *def2*), based on molecular marker–assisted selection (MAS) and HR test to TSWV NSm infiltration. (**E**) Fine-mapping of *RTSW* and *DEF2* loci using the indicated markers in five TSWV-resistant plants with no deformity. (**F**) Representative photographs of a TSWV-resistant plant with no deformity harboring *RTSW* only (K326-*RTSW*; #12 as an example) and the wild type (WT), K326, at the flowering stage. Scale bars, 10 cm. (**G**) Assessment of agronomic traits in plots for K326-*RTSW* and K326 plants in 2021 and 2022—plant height, leaf number, length of 14th leaf, width of 14th leaf, and dry weight of 10 leaves—using at least 10 randomly selected plants per trait. For box plots, boxes indicate the 25th to 75th percentile, whiskers indicate the full data range, center lines indicate the median, and dots indicate individual data points. Statistical significance of differences between K326-*RTSW* and K326 was determined using unpaired two-sided Student’s *t* tests, with the *P* values indicated accordingly.

To investigate the genetic basis of the *RTSW* and *DEF* loci, we generated a BC_6_F_2_ population by backcrossing a BC_5_F_3_ homozygous line (derived from repeated backcrossing of Polalta to the recurrent parent *N. tabacum* cultivar K326) to K326 and allowing self-pollination (Fig. 2A). Phenotypic segregation analysis of 454 BC_6_F_2_ plants revealed a 3:1 resistant:susceptible ratio (χ^2^_3:1_ = 0.9344, df = 1, *P* = 0.334; 319 resistant:118 susceptible among 437 tested) for the TSWV *NSm*–triggered hypersensitive response (HR), consistent with monogenic inheritance of a dominant resistance allele. In contrast, plant deformity segregated in a 15:1 Mendelian ratio (χ^2^_15:1_ = 2.7964, df = 1, *P* = 0.094; 417 deformed:37 normal) in the BC_6_F_2_ plants, shifting to 12:1 in the full population, suggesting that the deformity phenotype is likely controlled by multiple loci (data S12). To map these loci, we performed bulked segregant analysis sequencing (BSA-seq) with >30-fold genome coverage on phenotype-specific pools.

For *RTSW*, we selected 40 deformed resistant (R pool) and 40 deformed susceptible (S pool) individuals (Fig. 2A). We mapped the BSA-seq reads in two ways: (i) to the diploid *N. alata* genome assembly, as the resistance locus originated from this species, and (ii) to the allotetraploid *N. tabacum* genome, to confirm the location of the introgressed segments. We identified a 21.1-Mb region on chromosome 3 of the *N. alata* genome assembly (43.7 to 64.8 Mb) that genetically links to the *RTSW* locus and is native to *N. alata* (Fig. 2B). Mapping the reads to the K326 *N. tabacum* genome (*33*) revealed two peaks, one in each subgenome, in the regions orthologous to *N. alata* (fig. S2, A to C). The genotype information across all markers of the corresponding region confirmed that the *RTSW* locus originated from *N. alata* chromosome 3 and was introgressed into the end of chromosome Nt07 (fig. S2E).

To map the *DEF* loci, we randomly selected 40 deformed individuals and 37 individuals of normal phenotype to form the *DEF* and *def* pools, respectively (Fig. 2A). BSA-seq analysis identified two peaks reflective of differences between the two pools: one on *N. alata* chromosome 4 and another overlapping with the *RTSW* locus on chromosome 3 (Fig. 2B). To eliminate interference from the *RTSW* locus, we selected 40 deformed susceptible individuals (*DEF1* pool) and compared them to the *def* pool composed of susceptible individuals with no deformity (Fig. 2A). We detected only one peak (*DEF1*), located between 0 and 8 Mb of *N. alata* chromosome 4, that appears to be introgressed into the orthologous telomeric region of *N. tabacum* chromosome Nt11 (Fig. 2B and fig. S2, A, B, D, and F). To investigate the second *DEF* locus, we selected 40 deformed individuals lacking *DEF1* based on genotyping with sequence-characterized amplified region (SCAR) markers to form the *DEF2* pool. The peak for *DEF2* overlapped with the *RTSW* locus (Fig. 2A and fig. S2A), which is consistent with the observed HR, characteristic of resistance (*20*), upon infiltration of TSWV *NSm* in all the deformed individuals lacking *DEF1* (data S13).

Considering the tight linkage of *RTSW* and *DEF2*, we screened an additional 1500 plants from another BC_6_F_1_ population [K326 × BC_5_F_2_ (*rtsw/RTSW*)] using SCAR markers for *N. alata* chromosome 3 at 44.2 and 64.6 Mb and identified three recombinant plants. Using additional SCAR markers and HR tests on those three recombinants, we narrowed down the *RTSW* locus to a 58- to 65.7-Mb interval on *N. alata* chromosome 3 (fig. S3, A and B). However, the plant with the shortest *RTSW* introgression segment (plant #46) still carried *DEF1* on chromosome 4, based on genotyping with the SCAR markers NaChr4_2M and NaChr4_8M specific for *DEF1* (fig. S3A). Because the *DEF1* and *RTSW-DEF2* loci originate from different *N. alata* chromosomes (4 and 3, respectively) and were introgressed into different *N. tabacum* chromosomes (11 and 7, respectively), we expected it to be possible to segregate *DEF1* from *RTSW-DEF2* in high-generation backcrosses, but this proved challenging. In the self-pollinated F_2_ progeny from plant #46, we obtained 50 plants with the *RTSW def1* genotype, all of which exhibited even stronger morphological deformities, lower fertility, and less fruit setting (Fig. 2C) than the other genotype groups. These results suggest an antagonistic epistatic interaction between *DEF1* and *DEF2* (fig. S3C). Therefore, to achieve resistance without growth defects, we attempted to genetically isolate *RTSW* by simultaneously segregating both *DEF1* and *DEF2*.

**Fig. 3.**
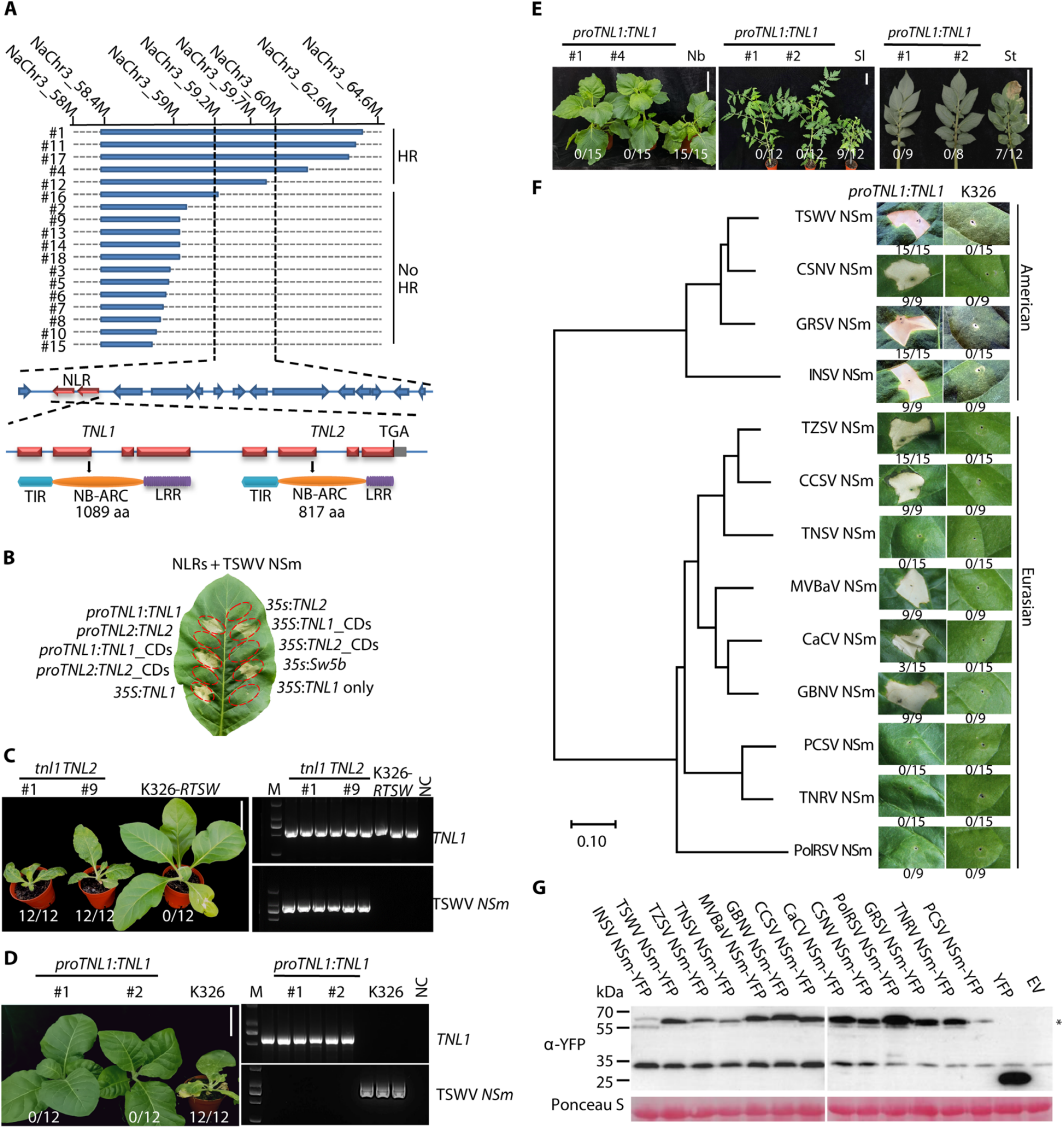
Characterization of the *RTSW* locus and the broad-spectrum resistance it conveys. (**A**) Fine-mapping of *RTSW*. The introgressed segments from *N. alata* shown as blue bars. The locus was narrowed down to two NLR candidate genes, *TNL1* (with intact four exons and functional domains) and *TNL2* (with premature TGA stop codon), using additional markers (top) and 18 recombinant plants (marked as #). HR test outcomes are marked. aa, amino acid. (**B**) Transient coexpression of *TNL1/TNL2* variants with TSWV *NSm* in WT K326. The constitutive cauliflower mosaic virus 35*S* promoter (35*S*) or native promoter [*proTNL1* (1875 bp) and *proTNL2* (2117 bp)] was instrumental in driving full-length genes (*TNL1* or *TNL2*) or their coding sequences (*CDs*). The 35*S*:*Sw-5b* construct was used as a positive control, and 35*S*:*TNL1* was tested for autoimmunity. (**C**) *tnl1* mutant lines generated through CRISPR-Cas9–mediated gene editing were tested for TSWV resistance alongside their parental K326-*RTSW* line. Genomic DNA and total RNA were extracted for polymerase chain reaction (PCR) confirmation of the *TNL1* genotype (*TNL1*) and TSWV presence (TSWV *NSm*). M, marker; NC, negative control. (**D**) Introduction of the *proTNL1*:*TNL1* construct into susceptible K326 conferred high resistance to TSWV in two randomly selected homozygous lines. Photographs were taken at 14 days postinoculation (dpi). (**E**) Transgenic *proTNL1*:*TNL1* lines in *N. benthamiana* (Nb), *S. lycopersicum* (Sl), and *S. tuberosum* (St) exhibit resistance to TSWV. (**F**) Induction of cell death by NSm proteins from 13 orthotospoviruses in the leaves of *RTSW* transgenic (*proTNL1*:*TNL1*) plants. The phylogenetic tree was reconstructed on the basis of NSm amino acid sequences. Photographs were taken at 3 dpi. The numbers under each image show the ratio of leaves showing HR to total infiltrated leaves. CSNV, chrysanthemum stem necrosis orthotospovirus; GRSV, groundnut ringspot orthotospovirus; GBNV, groundnut bud necrosis orthotospovirus; TNRV, tomato necrotic ring orthotospovirus; PolRSV, polygonum ringspot orthotospovirus. (**G**) Immunoblot detection of 13 yellow fluorescent protein (YFP)–tagged NSm proteins in (F). Ponceau S staining confirms equal loading. The asterisks indicate the specific band with the size predicted for NSm-YFP. The ratios in (C) to (E) refer to symptomatic:inoculated plants. Scale bars, 10 cm. EV, empty vector.

To this end, we screened a very large BC_7_F_1_ population of more than 160,000 seedlings derived from a cross between K326 and plant #46 (a BC_6_F_1_ plant with the genotype *rtsw/RTSW* and *def1/DEF1*) (Fig. 2D and fig. S4, A to C). We removed all deformed individuals and genotyped the remaining 12,000 morphologically normal plants (fig. S4D), culminating in 18 plants with recombination events within the *RTSW-DEF2* introgressed segment (details in Fig. 3A, Supplementary Text, and fig. S4E). Of these 18 plants, 5 exhibited resistance, based on HR tests (Fig. 2E and fig. S4, F and G). We selected individual #12, which harbored the shortest *RTSW* introgressed segment and exhibited exceptional resistance, for our resistance breeding program (Fig. 2F and fig. S5, A and B). Field evaluations demonstrated that the near-isogenic line K326-*RTSW*, derived from plant #12, displays agronomic traits (plant height, leaf number, dimensions, and dry weight) comparable to those of control K326 in field plots without tobacco spotted wilt disease (TSWD; Fig. 2G and fig. S5C). In fields naturally affected by TSWD, all K326-*RTSW* plants remained free of symptoms, whereas 20.4% of K326 plants died from TSWD infection (fig. S5, D and E).

### RTSW is a TNL receptor conferring broad orthotospovirus resistance

To fine-map *RTSW*, we genotyped all 18 recombinant plants with additional markers and narrowed the candidate region to an 800-kb interval between 59.2 and 60 Mb on *N. alata* chromosome 3 (Fig. 3A and fig. S5A) containing 15 predicted genes (data S14). Two of these are annotated as *N*- or *Target of AvrB operation1*–like resistance genes, encoding typical TNL receptors, and were thus designated *TNL1* and *TNL2*. While TNL1 contains intact TIR, NB, and LRR domains in its deduced 1089 amino acid sequence, TNL2 has a premature stop codon in its LRR domain, resulting in a truncated 875–amino acid protein (Fig. 3A).

To identify *RTSW*, we coinfiltrated TSWV *NSm* with constructs for *TNL1* or *TNL2* into K326 leaves. Only constructs harboring *TNL1*, not *TNL2*, triggered HR, suggesting a role for TNL1 in resistance (Fig. 3B). We then generated *tnl1*, *tnl2*, and *tnl1 tnl2* mutants in the K326-*RTSW* background via clustered regularly interspaced short palindromic repeats (CRISPR)/CRISPR-associated nuclease 9 (Cas9)–mediated gene editing (fig. S6, A and B). The *tnl1* mutant and the *tnl1 tnl2* double mutant produced no HR upon TSWV *NSm* infiltration, confirming the critical function of TNL1 (fig. S6C). Moreover, self-pollinated *tnl1* mutant plants showed complete susceptibility to TSWV (Fig. 3C). We also generated stable transgenic lines in susceptible K326 with *TNL1* controlled by its native promoter, which showed resistance to TSWV infection (Fig. 3D). Last, transgenic introduction of *TNL1* in other Solanaceae crops resulted in TSWV resistance in *Nicotiana benthamiana*, tomato (*S. lycopersicum*), and potato (*S. tuberosum*), with no virus detected in systemic leaves of inoculated transgenic plants (Fig. 3E and fig. S7). These findings unequivocally identify *TNL1* as the *RTSW* gene [National Center for Biotechnology Information (NCBI) accession no. PQ072337].

To assess the efficacy of *RTSW* in conferring resistance to other orthotospoviruses beyond TSWV, we conducted inoculation assays using *proTNL1:TNL1* transgenic tobacco plants and control plants with American- and Eurasian-type orthotospoviruses. While control plants were infected, transgenic *proTNL1:TNL1* plants showed marked resistance, particularly to impatiens necrotic spot orthotospovirus (INSV), tomato zonate spot orthotospovirus (TZSV), pepper chlorotic spot orthotospovirus (PCSV), calla lily chlorotic spot orthotospovirus (CCSV), mulberry vein banding–associated orthotospovirus (MVBaV), and capsicum chlorosis orthotospovirus (CaCV) (fig. S8). We then synthesized 13 *NSm* genes from orthotospoviruses known to infect tobacco and infiltrated them individually into *proTNL1:TNL1* transgenic and control tobacco plants. The NSm of all American-type and some Eurasian-type orthotospoviruses induced cell death in the *proTNL1:TNL1* transgenic plants, confirming the broad recognition of NSm by TNL1 (Fig. 3F). Immunoblotting analysis confirmed the accumulation of all viral NSm proteins (Fig. 3G). The results identify *RTSW* as a gene conferring broad-spectrum resistance against both American- and Eurasian-type orthotospoviruses, which infect a wide range of crops.

### Molecular and functional characterization of RTSW

**Fig. 4.**
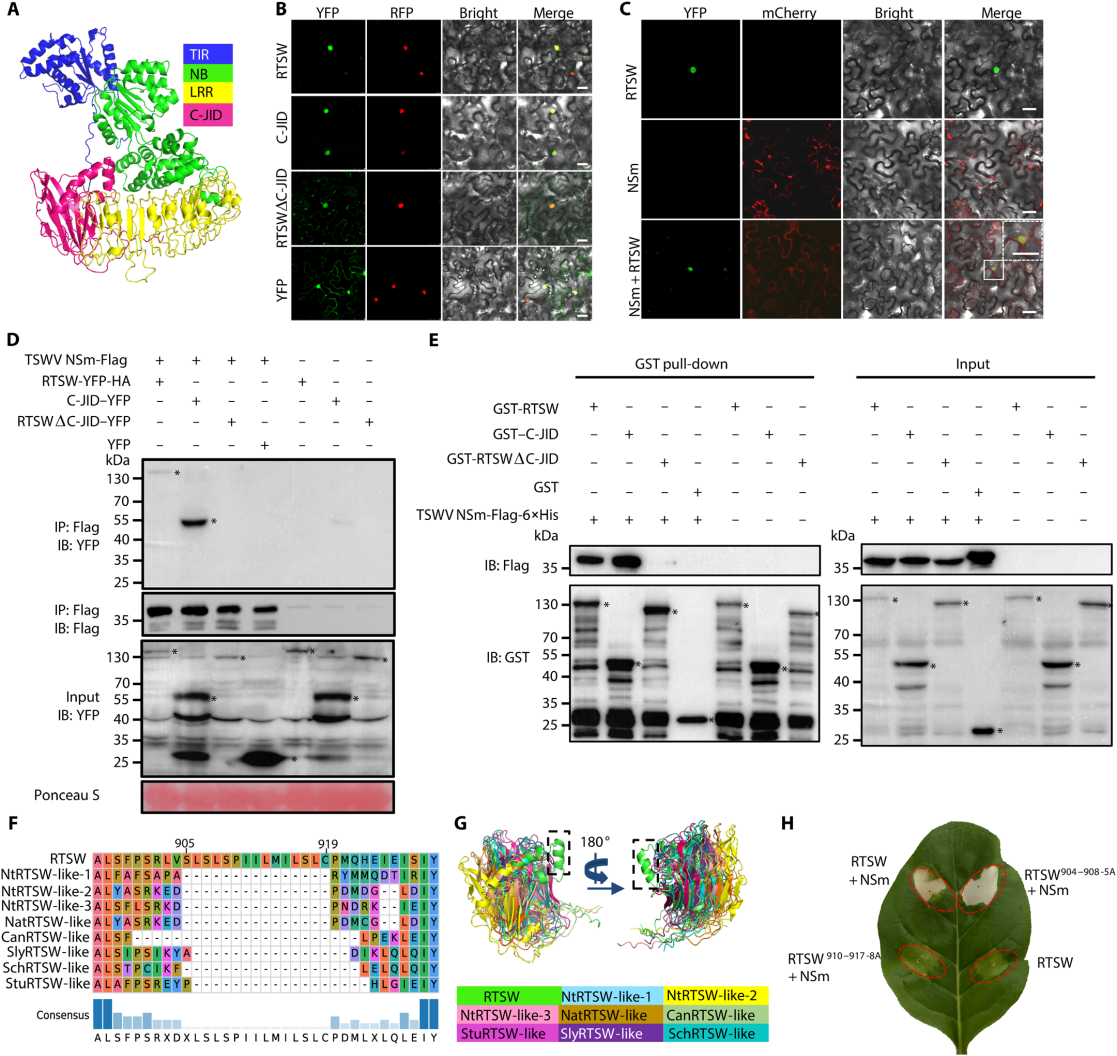
Molecular characterization of RTSW. (**A**) Predicted 3D structure of RTSW. (**B**) Subcellular localization of full-length RTSW, individual domains, and truncations. YFP-tagged constructs were coexpressed with nuclear marker H2B-RFP in *N. benthamiana* leaves. Fluorescence (YFP: green; RFP: red) imaged at 24 hours post inoculation by confocal microscopy. Scale bars, 25 μm. (**C**) Colocalization of RTSW-YFP (green) and NSm-mCherry (red) in *N. benthamiana*. Zoomed regions (top right) highlight nuclear overlap. Scale bars, 25 μm. (**D**) Coimmunoprecipitation (co-IP) assays. RTSW-YFP-HA, C-JID–YFP, or RTSWΔC-JID–YFP coexpressed with NSm-Flag in *N. benthamiana* and subjected to co-IP with anti-Flag antibodies. The blots were probed with anti-Flag or anti-YFP antibodies. Asterisks indicate the specific bands with the predicted size for the YFP fusion (top) or YFP alone (bottom). IB, immunoblot; IP, immunoprecipitation. (**E**) Glutathione *S*-transferase (GST) pull-down assay. Purified GST-RTSW, GST–C-JID, or GST-RTSWΔC-JID was incubated with NSm-Flag-6×His and immunoprecipitated on glutathione-Sepharose beads. The blots were probed with anti-GST and anti-Flag antibodies. The asterisks indicate the specific band with the predicted size for the GST fusion (top) or GST alone (bottom). (**F**) Partial protein sequence alignment between RTSW and its homologs from *N. tabacum* (NtRTSW-like), *N. attenuata* (NatRTSW-like), *S. lycopersicum* (SlyRTSW-like), *S. chilense* (SchRTSW-like), *C. annuum* (CanRTSW-like), and *S. tuberosum* (StuRTSW-like) (shown in fig. S10A). The unique motif (residues 905 to 919) in RTSW is highlighted. (**G**) Structural overlay of C-JID domains. The specific α helix of RTSW is indicated by the black dashed rectangles. (**H**) Transient coexpression of RTSW variants with NSm in K326. Amino acids in the α helix of RTSW and the N terminus of the α helix were replaced with alanine, yielding RTSW^910–917-8A^ and RTSW^904–908-5A^, respectively. RTSW alone was tested for autoimmunity. Experiments were repeated up to three times.

Using AlphaFold2 with conserved domain search (*34*), we predicted the 3D structure of RTSW, revealing typical TNL architecture with a conserved N-terminal TIR domain (amino acids 1 to 194), a central NB domain (amino acids 195 to 530), an LRR domain (amino acids 531 to 859), and a distinctive C-terminal jelly roll/immunoglobulin-like domain (C-JID; amino acids 860 to 1089) (Fig. 4A). To explore the protein’s subcellular localization, we fused the yellow fluorescent protein (YFP)–hemagglutinin (HA) tag to the C terminus of RTSW. Confocal microscopy analysis indicated that RTSW mainly localizes in the nucleus, as the YFP signal overlapped with that of the nuclear marker histone 2B (H2B) fused to the red fluorescent protein (H2B-RFP) (Fig. 4B). To delineate which domain is responsible for the nuclear localization of RTSW, we added YFP to the C terminus of each domain. We detected TIR-YFP, NB-YFP, and LRR-YFP in the cytoplasm and nucleus, while C-JID–YFP accumulated only in the nucleus, similar to full-length RTSW (Fig. 4B and fig. S9A). Conversely, deleting the C-JID (RTSWΔC-JID) resulted in YFP signals in the cytoplasm and nucleus (Fig. 4B). These results suggest that the C-JID is essential for the nuclear localization of RTSW.

To define the molecular basis of RTSW-mediated broad-spectrum orthotospovirus resistance, we investigated its interaction with the viral movement protein NSm through complementary biochemical and cellular assays. Colocalization assays in *N. benthamiana* epidermal cells coexpressing RTSW-YFP and NSm-mCherry revealed partial nuclear localization of NSm when coexpressed with RTSW, with spatial overlap confirmed by confocal microscopy (Fig. 4C). To test whether this colocalization reflects direct interaction, we performed coimmunoprecipitation (co-IP) assays. Coexpression of RTSW-YFP-HA with NSm-Flag (TSWV) or NSm-3 × Flag (TZSV) in *N. benthamiana* leaves showed that RTSW physically associates with NSm from both viruses (Fig. 4D and fig. S9B). Notably, deletion of the C-JID in RTSW (RTSWΔC-JID) abolished this interaction (Fig. 4D), indicating the C-JID is essential for NSm recognition. To further resolve the interaction mechanism, glutathione *S*-transferase (GST) pull-down assays demonstrated that GST-tagged RTSW and its isolated C-JID domain, but not RTSWΔC-JID, directly bound NSm from TSWV and TZSV (Fig. 4E and fig. S9C). Collectively, these results suggest that the C-JID of RTSW is responsible for the direct recognition of orthotospovirus NSm.

*RTSW* is the sole TNL-type TSWV resistance gene identified within the Solanaceae family. To investigate this unique feature, we aligned sequences of representative TNL proteins with high similarity to RTSW from various TSWV-susceptible species, including *N. tabacum*, *Nicotiana attenuata*, *S. lycopersicum*, *Solanum chilense*, *C. annuum*, and *S. tuberosum*. Compared to the relatively conserved TIR, NB, and LRR domains, the C-JID sequence showed high diversity (fig. S10A). Notably, a 15–amino acid motif (amino acids 905 to 919) occurred only in the C-JID of RTSW (Fig. 4F and fig. S10A). AlphaFold2 structure predictions suggested that residues 910 to 917 within the C-JID of RTSW form a prominent α helix (Fig. 4G and fig. S10B). We hypothesized that this α helix is crucial for NSm recognition. Accordingly, we replaced all of the amino acids at positions 910 to 917 with alanines (RTSW^910–917-8A^) and replaced the five amino acids at the N terminus of the α helix with alanines (RTSW^904–908-5A^) as control. In contrast to the HR phenotype observed when *RTSW* or *RTSW*^*904*–*908-5A*^ was coinfiltrated with *NSm*, coinfiltration of *RTSW*^*910*–*917-8A*^ did not trigger HR in the presence of NSm (Fig. 4H). Thus, the α helix is a key element in RTSW recognition of NSm and the subsequent activation of RTSW-mediated plant immunity.

### Evolution of RTSW

**Fig. 5.**
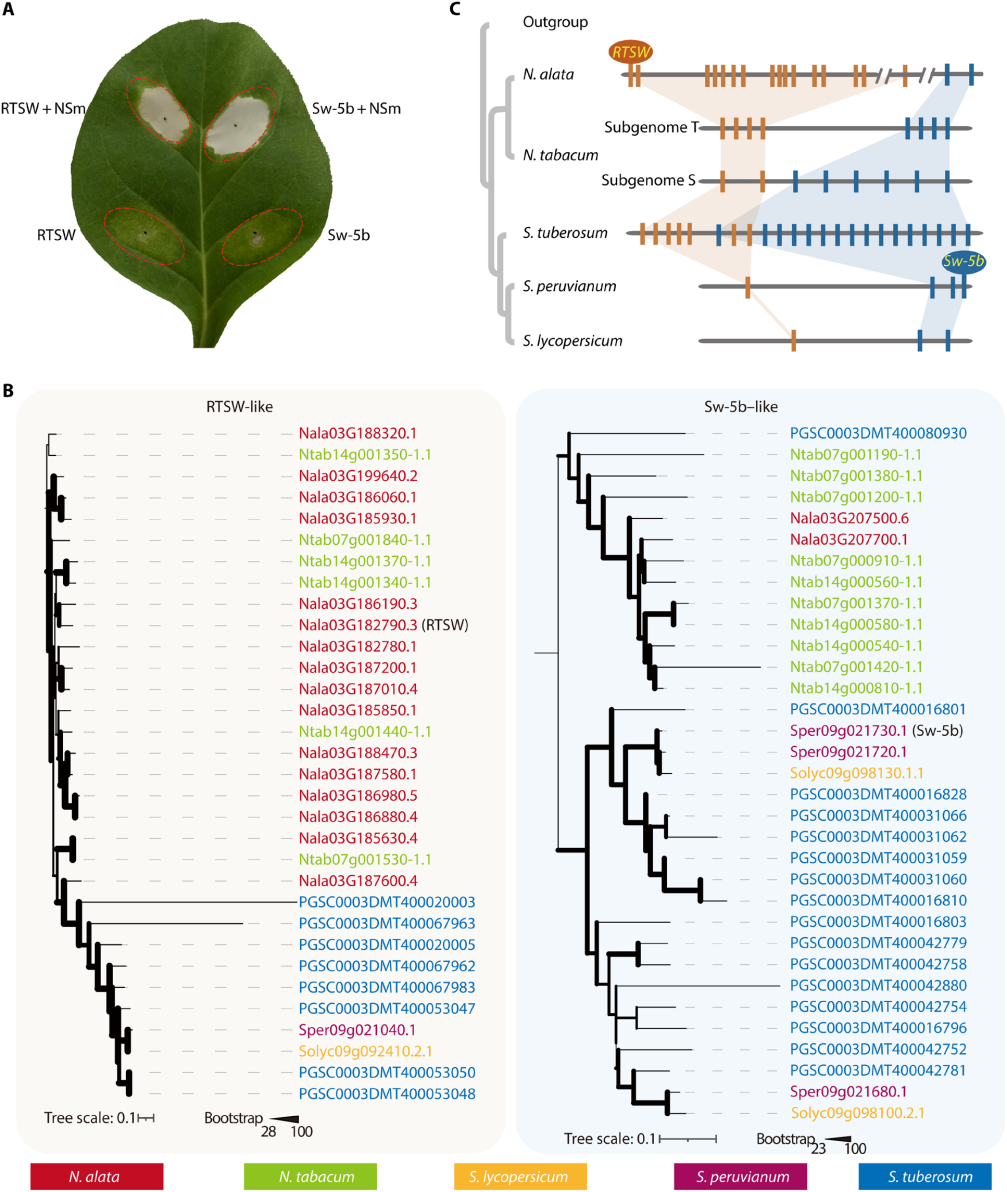
Functional and evolutionary roles of the *RTSW* and *Sw-5b* genes. (**A**) RTSW and Sw-5b recognize NSm of TSWV. TSWV *NSm* was coinfiltrated with RTSW (RTSW + NSm) or Sw-5b (Sw-5B + NSm) in K326 leaves; *RTSW* or *Sw-5b* was infiltrated alone as a control. The experiments were repeated up to three times with similar results. (**B**) Phylogeny of RTSW-like and Sw-5b–like proteins within the syntenic regions of the *N. alata*, *N. tabacum*, *S. lycopersicum*, *S. peruvianum*, and *S. tuberosum* genomes. NLRs from different species are color coded according to the legend (bottom). Branch thickness represents the level of support from 1000 bootstrap replicates. (**C**) Diagram of the proposed evolutionary trajectory and functional retention of the *RTSW* (dark orange) and *Sw-5b* (blue) clusters.

We previously showed that Sw-5b from *S. peruvianum* and RTSW from *N. alata* recognize the same NSm Avr from TSWV (Fig. 5A) but via different domains/motifs (*20*). Here, we demonstrated that *RTSW* is a TNL, while *Sw-5b* is a CNL resistance gene. Phylogenetic analysis revealed an expansion of the TNL gene family on *N. alata* chromosome 3 (Figs. 1, C and D, and 5B), leading to closely positioned tandem TNL genes, one of which is *RTSW*. Conversely, a CNL tandem gene duplication on *S. peruvianum* chromosome 9 resulted in the genesis of *Sw-5b* (Fig. 5B). Notably, we found that *N. alata* chromosome 3 and *S. peruvianum* chromosome 9 are syntenic (fig. S11A). Microsynteny analysis further showed the ancestral positioning of the *RTSW* and *Sw-5b* clusters, within 600 kb on chromosome 9 of *S. peruvianum* and 9 Mb on chromosome 3 in *N. alata* (Fig. 5C and fig. S11A). By contrast, another CNL resistance gene, *Tsw*, whose encoded protein recognizes the NSs open reading frame of TSWV (*11*), is located on chromosome 10 of *C. chinense* (*9*) and has a region syntenic with *N. alata* chromosome 1 (fig. S11B). The proximity of the ancestral loci that evolved into distinct NLR genes and conferred resistance to the same virus with the same Avr factor recognition after speciation is intriguing and merits further investigation.

## DISCUSSION

*N. alata* is a South American native and widely cultivated ornamental plant with distinctive traits that make it an attractive model for academic and industrial research (*35*–*40*). Moreover, *N. alata* is the closest relative of cultivated tobacco that is resistant to diseases such as TSWV, tobacco mosaic virus, and black root rot (*22*, *41*). We present here a chromosome-anchored genome assembly of *N. alata*, an assembled genome of diploid *Nicotiana* section Alatae, with a basic chromosome number of *n* = 9 (*39*), differing from the common *n* = 12 of most Solanaceae plants. Although we focused our study on resistance, this de novo *N. alata* genome assembly should facilitate broader exploration of important traits in this species.

The TSWV resistance trait of *N. alata* was identified in 1969 and introduced into cultivated *N. tabacum*, leading to the development of the Polalta breeding line in the 1980s (*19*, *21*). Notably, this resistance was always accompanied by plant deformity (fig. S4A) (*22*, *23*). Here, we mapped both resistance and deformity traits observed in Polalta to the *N. alata* genome using BSA-seq (Fig. 2), identifying the resistance locus *RTSW* on chromosome 3 and two deformity loci on chromosomes 4 (*DEF1*) and 3 (*DEF2*), the latter being tightly linked to *RTSW*. In addition to this tight linkage, plants lacking *DEF1* but having *DEF2* were even more deformed and sterile than other *DEF* genotypes (Fig. 2C), making stepwise segregation of deformity from resistance next to impossible. The genetic complexity of deformity might have historically hindered the separation of resistance and deformity traits in introgressed *N. tabacum* lines.

Simultaneous segregation of *DEF1* and *DEF2* away from *RTSW* is therefore a numbers game, which we faced by screening >160,000 plants from a BC_7_F_1_ segregating population. We genotyped only plants devoid of deformity via marker-assisted selection, yielding five plants carrying *RTSW* and lacking both *DEF1* and *DEF2* (fig. S4). We demonstrated that these *RTSW* plants have the same capacity to resist the virus as Polalta and *N. alata* (figs. S4G and S5B), with similar agronomic traits to K326 in the field (Fig. 2G and fig. S5C). The K326-*RTSW* cultivar can be directly used in resistance breeding programs to improve the TSWD resistance of *N. tabacum* cultivars.

Although we did not identify the genes underlying *DEF1* and *DEF2*, they appear to map to the two largest NLR gene clusters in the *N. alata* genome on chromosomes 3 and 4, containing 16 and 20 predicted NLR genes, respectively. We propose a model to elucidate the genetic architecture of resistance and deformity traits in Polalta (fig. S3C). We hypothesize that regulatory genes present in the *DEF1* locus can finely tune gene expression, protein abundance, or modifications—potentially of NLRs—in the *DEF2* locus or both the *DEF1* and *DEF2* loci. In the absence of *DEF1*, the dysregulation of genes within *DEF2* plants leads to severe developmental defects. The genes responsible for this regulation and the underlying molecular mechanisms warrant further investigation.

Using 18 recombinant individuals from the >160,000 BC_7_F_1_ segregating population, we narrowed *RTSW* down to two TNL genes in an 800-kb region, of which only *TNL1* was necessary and sufficient for resistance (Fig. 3A). We also demonstrated that *RTSW* confers the broadest resistance to orthotospoviruses among all known resistance genes (*9*, *14*, *30*). RTSW-mediated resistance was effective not only against all American types but also against several Eurasian types of orthotospoviruses; moreover, RTSW-triggered immunity proved effective in all tested Solanaceae crops (Fig. 3, E and F). Last, we revealed the primary molecular mechanism of recognition between RTSW and its Avr factor, NSm, which involves an α helix in the C-JID of RTSW (Fig. 4).

Exploring microsynteny between expanded regions in *N. alata* and other high-quality Solanaceae genomes, we discovered that a previously reported TSWV resistance gene in *S. peruvianum* (*Sw-5b*) originated in close proximity to the ancestral region of *RTSW* in *N. alata* (Fig. 5C and fig. S11). *RTSW* and *Sw-5b* are not orthologous, as one encodes a TNL and the other a CNL, defining this independent evolution of resistance to the same virus by recognizing the same *Avr* gene from the same ancestral region as an interesting coincidence. This fact may have misled many researchers attempting to identify *RTSW* by looking for an ortholog of *Sw-5b*. The close proximity of their respective ancestral loci that have evolved into distinct NLR genes, each targeting the same pathogen molecule, is a fascinating aspect of evolutionary biology. This observation suggests a model of parallel evolution in which similar selective pressures, specifically TSWV infection in this case, have driven the expansion and diversification of NLR genes in similar genetic landscape contexts in related species. The detailed evolutionary trajectory of these genes, including the specific mutations and selective events that have shaped their recognition capabilities, warrants exploration. This knowledge could inform strategies for breeding plants with enhanced and durable resistance to pathogens. In summary, our work provides an important resistance gene that can be used for crop improvement and a case study to explore the robust and effective mechanism of broad resistance conferred by TNLs.

## MATERIALS AND METHODS

### Plant and virus materials

*N. alata* (accession no. “PI42334”), used for genome sequencing, was provided by the US Department of Agriculture, Agricultural Research Service (ARS), National Genetic Resources Program. The breeding line Polalta was supplied by R. Lewis from North Carolina State University. Backcrossing of Polalta to the recurrent tobacco (*N. tabacum*) parent “K326” was performed to generate different generations of BC lines, with selection for RTSW resistance applied at each generation to eliminate nontarget DNA regions.

The orthotospoviruses used in this study were TSWV, INSV, TZSV, tomato necrotic spot orthotospovirus, PCSV, CCSV, MVBaV, and CaCV. All viruses were isolated from diseased plants growing in the Yunnan province of China and propagated on *N. benthamiana* plants in a greenhouse. For virus inoculation, crude saps from the leaves of infected *N. benthamiana* plants were rubinoculated onto the leaves of healthy plants as described (*20*).

### Estimation of genome size and heterozygosity

The *N. alata* genome size was estimated using flow cytometry and *k*-mer analysis. Flow cytometry followed a previously described method (*42*), using a BD FACSCalibur flow cytometer (BD Biosciences, California, USA) with propidium iodide staining. Tomato (*S. lycopersicum*) “M82,” with a known genome size of ~0.88 Gb, served as an internal standard (*43*). The *k*-mer analysis was conducted using Jellyfish v.2.2.5 with Illumina sequencing data as input (*44*), with error correction performed using Trimmomatic-0.32 (*45*) and FastQC (www.bioinformatics.babraham.ac.uk/projects/fastqc/). GenomeScope was used for estimating genome size, repeat content, and heterozygosity, as described (*46*).

### Genome sequencing

High–molecular weight genomic DNA was extracted from *N. alata* seedlings following a 72-hour dark treatment. Several libraries were constructed with insert sizes ranging from 450 bp to 10 kb and sequenced on Illumina platforms. A 10x Genomics linked-read library was sequenced to achieve 75-fold coverage (BGI, Shenzhen, China). Clean data were obtained by removing sequencing errors, polymerase chain reaction (PCR) duplicates, and adapters. For Hi-C sequencing, a library was constructed and sequenced on an Illumina HiSeq X Ten instrument. Raw Fastq files were processed using HiC-Pro (v2.11.1) to remove duplicates and assess data quality (*47*).

### Genome assembly

A de novo whole-genome assembly was performed using DeNovoMAGIC3 software (NRGene, Nes Ziona, Israel), optimized for polyploid and heterozygous genomes (*48*, *49*). The 10x Genomics data were used to phase and validate haplotype scaffolds. The initial assembly resulted in a contig N50 size of 161.07 kb and a scaffold N50 size of 10.17 Mb (data S2). Hi-C reads were mapped to the nonphased genome assembly using Juicer (v1.5.6) (*50*), and chromosome-length assemblies were generated with the 3D DNA (v180922) pipeline (*51*). Manual refinement was performed using Juicebox (v1.13.01) (*52*), and a final pseudochromosome-level genome assembly was obtained with nine pseudomolecules using 3D DNA (*51*). The BUSCO score was calculated against the v3 eudicots_odb10 list of orthologs to evaluate the quality and completeness of the genome assembly (*53*).

### RNA library preparation and sequencing

Plant tissue (root, leaf, stem, and flower) samples from *N. alata* were collected and subjected to total RNA extraction using an RNAiso Pure RNA Isolation Kit (Takara, Japan). RNA quality was assessed on a NanoVue Plus spectrophotometer (GE Healthcare, NJ, USA). RNA samples were used to generate RNA sequencing (RNA-seq) libraries, which were sequenced on an Illumina HiSeq X instrument (BGI) as paired-end reads.

### Genome annotation

Repeat sequences within the *N. alata* genome were annotated using RepeatMasker (v4.0.8), RepeatProteinMask, and de novo prediction tools such as LTR_FINDER and RepeatModeler (*54*–*56*). Tandem repeats were predicted using Tandem Repeats Finder (*57*), and the identified repeats were classified using RepeatClassifier (https://github.com/Dfam-consortium/RepeatModeler/blob/master/RepeatClassifier). Protein-coding genes were predicted using a combination of homologous comparison, ab initio prediction, and RNA-seq–based annotation. Searches for homologs were performed using BLASTX 2.11.0+ (*58*) (using the translated *N. alata* genome as a query) against reference protein sequences from the Solanaceae Genomics Network (https://solgenomics.net/). De novo prediction used tools such as Augustus, GlimmerHMM, SNAP, GeneScan, FGENESH, and GeneID (*59*–*63*). RNA-seq reads were assembled using Trinity v2.14.0 (*64*) and aligned against the *N. alata* assembly using PASA v2.3.3 (*65*). High-confidence gene models were predicted using EVidenceModeler v1.1.1 and the MAKER pipeline (*65*, *66*). Functional gene annotation was performed using EggNOG-mapper (*67*) and HMMER (*68*) to obtain clusters of orthologous groups of proteins, evolutionary genealogy of genes: non-supervised orthologous groups, Gene Ontology, and Kyoto Encyclopedia of Genes and Genomes pathway information (*69*). Noncoding RNA annotation included tRNA prediction using tRNAscan-SE (*70*) and ribosomal RNA, small nuclear RNA, and microRNA annotation using Infernal and the Rfam database (*71*).

### Whole-genome sequencing–based BSA-seq

Genomic DNA was isolated from young leaves of BC_6_F_2_ individuals, with 40 individuals for each of the resistant (R), susceptible (S), *DEF*, *DEF1*, and *DEF2* pools and 37 for the *def* pool; genomic DNA from the two parental lines was also prepared, using a plant genomic DNA extraction kit (TIANGEN, Beijing, China). Equal amounts of genomic DNA from each individual were mixed to prepare each pool. Paired-end sequencing libraries were constructed from the pooled genomic DNA samples and the parental lines and sequenced on an Illumina HiSeq X10 instrument (Illumina) as paired-end 150-bp reads. High-quality clean 150-bp reads were obtained after removing reads containing adapters, more than 10% unidentified nucleotides (N), and quality scores (*Q*) ≤ 20 across more than 50% of the bases. Sequencing data were aligned to the previously published *N. tabacum* K326 genome and to our *N. alata* genome assembly using Burrows-Wheeler Aligner (BWA) (version 0.7.17-r1188) as described (*72*). SAMtools (version 1.16.1) was used to convert alignment files into binary alignment/map format and remove potential PCR duplicates. Variant calling and single-nucleotide polymorphism (SNP) filtering for each sample were performed using Genome Analysis Toolkit (GATK) (version 4.2.4.1) (*73*), yielding a total of 308,290 and 1,254,294 high-quality SNPs against the *N. alata* and K326 genomes, respectively. To enhance reliability, Euclidean distance (ED) analysis was used for BSA-seq data (*74*). ED was calculated at each SNP location using the formula with the ED value raised to the fourth power.

### Molecular marker and genetic map development

To validate the BSA-seq results, specific SCAR markers for the *RTSW-DEF2* and *DEF1* loci were developed on the basis of regions on chromosomes 3 and 4 of the *N. alata* genome assembly (data S15). In addition, a previously established simple sequence repeats (SSR) library (*75*) was screened, identifying nine SSR markers on Nt07 and four on Nt11 in the K326 genome that were tightly linked to the *RTSW-DEF2* and *DEF1* loci, respectively (data S15). All markers were validated in the corresponding population, and genetic maps were constructed using JoinMap 4 with the maximum-likelihood algorithm.

### Deformity assessment and screening

For phenotypic analysis, a BC_7_F_1_ population was obtained by crossing K326 (recurrent parent; *rtsw-def2/rtsw-def2* and *def1/def1* genotype) with plant #46 (*RTSW-DEF2/rtsw-def2* and *DEF1/def1* genotype), which was heterozygous for the *def1* and *def2* alleles. The BC_7_F_1_ population was grown in 1100 floating plates with approximately 150 seeds per plate, totaling more than 160,000 seedlings subjected to screening. Abnormal leaf morphologies, observable from the cotyledon to the six-true-leaf stages, were assessed over five rounds. Morphological abnormalities included thickened and ribbon-shaped leaves, irregular venation, and dwarfism. After rigorous screening, 12,000 completely normal seedlings were selected for molecular marker screening.

### Marker-associated selection and fine mapping

In identifying plant #46, genomic DNA was extracted and subjected to PCR with primer pairs specific for markers NaChr3_44.2M and NaChr3_64.6M. Recombination events were indicated by the absence of a signal for either marker. Genomic DNA was extracted from the 12,000 normal seedlings, and the molecular markers NaChr3_58.4M and NaChr3_64.6M, flanking the resistance segment introgressed into plant #46, were used to screen for recombination events, indicated by the presence of at least one positive marker. Additional SCAR markers, NaChr3_60M and NaChr3_62.6M, were developed for mapping *RTSW* and *DEF2* based on the *N. alata* genomic sequence. To fine-map *RTSW*, additional SCAR markers NaChr3_59M, NaChr3_59.2M, and NaChr3_59.7M were designed and used to genotype all 18 recombinants, delimiting the *RTSW* gene to an 800-kb region flanked by markers NaChr3_59.2M and NaChr3_60M. PCR products were validated using the ZAG DNA Analyzer System (Agilent Technologies, USA).

### Plasmid construction

The plasmids p2300S-YFP, p2300S-3 × Flag, and p2300S-Sw-5b, and those fused with TSWV-NSm, TZSV-NSm, and INSV-NSm for C-terminal YFP fusions, were previously described (*14*, *20*). For other NSm proteins, the full-length coding sequences encoding them were custom-synthesized (GenScript, Jiangsu, China), sequenced, and cloned into the Bam HI–digested p2300-35S-YFP vector. To create full-length *TNL1* and *TNL2* genes or coding sequences driven by their native promoters, PCR products were amplified from Polalta genomic DNA (for promoter fragments: 1875 bp upstream of the *TNL1* translation start site and 2117 bp upstream of *TNL2*) and reverse-transcribed cDNA (for coding sequences), respectively. These fragments were cloned into Sac I– and Xba I–digested pHellsgate8 using homologous recombination (*72*), generating constructs including *proTNL1:TNL1* and *proTNL2:TNL2* (full-length promoter and gene), *proTNL1:TNL1_CDs*, and *proTNL2:TNL2_CDs* (promoter and coding sequence). Overexpression constructs for *TNL1* or *TNL2* were generated by cloning the full-length coding sequences of *TNL1* and *TNL2* individually into Xho I– and Xba I–digested pHellsgate 8. For subcellular localization, YFP tags were added to the C terminus of different RTSW domains, and the encoding sequences were inserted into p2300S-YFP. For co-IP assays, YFP tags were added to different RTSW domains and variants, and Flag or 3×Flag tags were added to TSWV-NSm or TZSV-NSm. For GST pull-down assays, the full-length coding sequences of RTSW, C-JID, and RTSW without the C-JID (RTSWΔC-JID) domain were cloned into pGEX-2TK, and a Flag tag was fused to TSWV-NSm or TZSV-NSm and cloned into pET28a. The primers for all plasmid construction are listed in data S15.

To create the alanine substitution variants of RTSW specifically targeting the α-helical region (residues 910 to 917, designated as RTSW^910–917-8A^) and the N terminus (residues 904 to 908, designated as RTSW^904–908-5A^), the *proTNL1*:*TNL1* plasmid was double digested using the restriction enzymes Avr II and Sna BI, resulting in the precise excision of a 1101-bp fragment from the fourth exon of *RTSW*, encoding the entire C-JID domain. Custom-synthesized DNA fragments designed to include the desired alanine substitutions (either 910–917-8A or 904–908-5A) were verified by Sanger sequencing to ensure accuracy. These fragments were then ligated into the linearized *proTNL1*:*TNL1* construct, thereby integrating the mutations into the *RTSW* sequence.

### RTSW rapid identification, knockout, and complementation

Cultures of *Agrobacterium tumefaciens* strain EHA105 carrying single constructs with candidate genes, *TSWV-NSm* or *TZSV-NSm*, were used for coinfiltration into K326 plants. Bacterial culture and preparation were conducted as previously described (*20*). Each construct was resuspended in infiltration buffer [10 mM MES (pH 5.6), 10 mM MgCl_2_, and 100 μM acetosyringone] to an optical density (OD_600_) of 1.0. The cell suspensions harboring *TSWV-NSm* or *TZSV-NSm* were mixed with various constructs at a 1:1 (v/v) ratio. To test for autoimmunity conferred by *TNL1*, the 35*S:TNL1* construct was infiltrated at an OD_600_ of 0.5. The development of an HR was observed 2 to 3 days postinfiltration. Each experiment was performed at least twice with a minimum of six independent replicates.

The K326-*RTSW* (#12) plant, harboring a single copy of the *RTSW* allele (*rtsw/RTSW*), was used as background for *TNL1* and *TNL2* knockout using our established Cas9-PF gene editing system (*76*). Given the high similarity between *TNL1* and *TNL2*, a single guide RNA (sgRNA) targeting a conserved region in the first exon was used to edit both genes individually or simultaneously. The sgRNA was synthesized (data S15) and cloned into the Bsa I–digested Cas9-PF vector as described (*76*, *77*). An *Agrobacterium* (strain EHA105) culture carrying the Cas9-PF-sgRNA construct was used to transform leaf disks of the K326-*RTSW* (#12) plant, yielding 30 positive T0 transgenic plants after selection for hygromycin resistance and regeneration of resistant calli. Gene editing events in transgenic tobacco plants were confirmed by Sanger sequencing of PCR products with the TNL1editTestF/R and TNL2editTestF/R specific primer pairs (data S15). Resistance was evaluated through *TSWV-NSm* infiltration–induced HR. Two randomly selected *tnl1* mutants with intact *TNL2* were self-pollinated, and the T1 generations of these edited plants were tested for resistance to TSWV. Each experiment was repeated up to three times, and each experimental repeat included six technical replicates.

For complementation studies, the *proTNL1*:*TNL1* construct was transformed into K326, *N. benthamiana*, tomato, and potato (*S. tuberosum*) plants using *Agrobacterium*-mediated leaf disk transformation. T0 plants, which were resistant to the application of kanamycin (100 μg/ml), were screened by PCR using specific primers (data S15). Two positive transgenic plants were randomly selected to obtain their T1 progeny, the resistance of which was evaluated with virus mechanical inoculation. Each experiment was repeated up to three times, and each experimental repeat included six technical replicates.

### Co-IP assays

Co-IP assays were performed as described (*14*). Total protein was extracted from *Agrobacterium*-infiltrated *N. benthamiana* leaves and incubated with anti-Flag (M2; Sigma-Aldrich, Missouri, USA) or anti–green fluorescent protein (ChromoTek, Hubei, China) nanobody agarose beads. The beads were washed six times with IP buffer [25 mM tris-HCl (pH 7.5), 1 mM EDTA, 150 mM NaCl, 10% (v/v) glycerol, 1 mM dithiothreitol, and 0.1% (v/v) Triton X-100] by centrifuging at 1000*g* and 4°C for 1 min. Protein samples were heated at 95°C for 5 min and separated by SDS–polyacrylamide gel electrophoresis (SDS-PAGE), transferred to polyvinylidene difluoride membranes, and incubated with anti–Flag–horseradish peroxidase antibodies (Sigma-Aldrich, catalog no. A8592; clone M2; 1:10,000) or anti-YFP (Sigma-Aldrich, catalog no. SAB4301138; 1:10,000). Blots were detected using an ECL Substrate Kit (Thermo Fisher Scientific, Massachusetts, USA), and protein loading was estimated by Ponceau S staining.

### Protein production and GST pull-down assays

Constructs pGEX-2TK-RTSW, pGEX-2TK-RTSWΔC-JID, pGEX-2TK–C-JID, pET28a-TSWV-NSm-Flag-6×His, and pET28a-TZSV-NSm-Flag-6×His were individually transformed into *Escherichia coli Rosetta* (DE3) strain. To express the recombinant protein, about 10 ml of each overnight culture was transferred to 1 liter of LB and incubated at 37°C until OD_600_ reached 0.6 to 0.8. Protein expression was induced with 0.1 mM isopropyl-β-d-thiogalactopyranoside for 16 hours at 20°C. Cells were lysed, and supernatants were collected for GST pull-down assays with glutathione-agarose beads as described (*15*). Assayed proteins were detected after SDS-PAGE and transferred to membranes by immunoblotting using anti-Flag or anti-GST antibodies.

### Confocal laser scanning microscopy

Tissue samples from *N. benthamiana* leaves expressing various *YFP* fusion constructs were collected and observed using a confocal laser scanning microscope. For confocal imaging, the excitation wavelength for YFP was set at 488 nm, and the emission was captured at 490 to 520 nm, and the excitation wavelength for RFP was set at 555 nm, and the emission was captured at 590 to 630 nm. RFP-H2B signals were used to mark the nucleus. The fluorescence signal was examined under a Zeiss LSM 710 confocal microscope (Zeiss) at 24 hours post–*Agrobacterium* infiltration.

### Structural prediction of RTSW and homologs

To predict the 3D structures of RTSW and its evolutionary homologs, BLASTp searches were initially conducted against the NCBI nonredundant protein database using the full-length RTSW protein sequence as the query, applying a stringent *E* value threshold (≤1 × 10^−20^) to identify high-confidence homologs. Sequences of representative species in Solanaceae meeting rigorous similarity criteria (≥70% amino acid sequence identity and ≥80% pairwise alignment coverage) were selected to ensure evolutionary conservation and structural relevance. The resulting RTSW-like protein set was subjected to AlphaFold2 structure prediction using the ColabFold v1.5 notebook (*34*). The structures with the highest predicted local distance difference test (pLDDT) scores were chosen as the most potentially accurate predictions. Proteins with existing AlphaFold-predicted structures in NCBI were directly retrieved for analysis. All predicted protein structures were aligned using PyMOL (www.pymol.org/pymol).

### Annotation and phylogenetic analysis of NLR genes

NLR genes from *C. chinense*, *C. annuum*, *S. lycopersicum*, and *S. tuberosum* were retrieved from the Angiosperm NLR Atlas (ANNA) database (*78*). NLR genes from *N. alata*, *N. tabacum* (*79*), and *S. peruvianum* (*43*) were annotated using a similar pipeline as described (*78*). Briefly, all protein sequences encoded by each genome were screened for the presence of an NB domain (Pfam: PF00931) using the HMM search with HMMER 3.0 (http://hmmer.org/) and default parameters. The identified NLR candidates were classified into TNL (with TIR domain), CNL (with CC domain), RNL, and unclassified NLR subclasses based on domain searches using their amino acid sequences. Amino acid sequences of NLR proteins and different subsets of NLR proteins were aligned using MAFFT v7.505. Poorly aligned regions were removed using trimAl. Phylogenetic analysis was performed using IQ-TREE (version 1.6.10) with the maximum-likelihood algorithm, including a best-fit model test and 1000 bootstrap replicates.

### Macrosynteny and microsynteny analyses

Macrosynteny and microsynteny analyses were performed using the Python-based tool MCscan [https://github.com/tanghaibao/jcvi/wiki/MCscan- (Python version)]. For the analysis of the *RTSW* and *DEF* loci, the *N. alata* genome from this study and the *N. tabacum* K326 genome (*33*) were used. For RTSW and Sw-5b evolution analysis, *N. alata*, *S. lycopersicum* (ITAG2.4), *S. tuberosum* (v4.03), *S. peruvianum* (*43*), and *N. tabacum* (*79*) were selected. Protein sequences and their corresponding gene model annotations were collected in Browser Extensible Data (BED) format. These data were analyzed to determine conserved gene order across the genomes using default parameters. Homologous regions between the selected Solanaceae species were identified by BLASTp searches (v 2.12.0+), and syntenic blocks were extracted for visualization.

### Field evaluation of agronomic and resistance traits

To evaluate the effect of the *RTSW* introgression on agronomic performance, K326 and K326-*RTSW* plants were cultivated in Yuxi, China, in 2021 and 2022. The assessment of field resistance was conducted in Chuxiong, China, in 2024. The trial was conducted under standard regional field management practices and organized in a randomized block design with three replicates. All genotypes were grown on floating plates in a greenhouse to obtain healthy seedlings in early March that were transplanted into the field 30 days after germination, with four to five leaves and heights of 5 to 7 cm. Plants were spaced 50 cm apart within rows and 100 cm between rows, resulting in a planting density of 15,000 to 16,500 plants per hectare. The following four quantitative traits were assessed and recorded in the non-TSWD field at 120 days posttransplanting: plant height (in centimeters), leaves per plant (in numbers), width of 14th leaf (in centimeters), and length of 14th leaf (in centimeters). For dry weight assessment, 10 leaves from the middle section of each plant were harvested and processed following standard tobacco curing methods. Samples for analysis were collected from three or four plants in the central row of each plot. To evaluate natural resistance, plants were grown in fields historically affected by TSWD. During the growing season, plants displaying TSWD symptoms were removed to prevent virus spread. The survival rate was assessed in fields before the initial harvest, with five randomly selected rows.

### Statistical analysis

Statistical analysis of all the data was performed using a two-sided Student’s *t* test in Microsoft Excel.
