# Supplementary material for: Identification of a gene conferring broad-spectrum orthotospovirus resistance in Solanaceae
Source: Sci Adv. 2025 Jun 18;11(25):eadw4333. doi: 10.1126/sciadv.adw4333 (PMC12175906; doi:10.1126/sciadv.adw4333)
Supplement: Supplementary file 1 — Supplementary Text Figs. S1 to S11 Legends for data S1 to S15 Data S16 [file sciadv.adw4333_sm.pdf]

Science Advances
AAAS

Supplementary Materials for

Identification of a gene conferring broad-spectrum orthotospovirus resistance in Solanaceae

Yong Liu *et al.*

Corresponding author: Polina Yu. Novikova, pnovikova@mpipz.mpg.de; Changjun Huang, cjhuang@zju.edu.cn

Sci. Adv. **11**, eadw4333 (2025)
DOI: 10.1126/sciadv.adw4333

The PDF file includes:

Supplementary Text
Figs. S1 to S11
Legends for data S1 to S15
Data S16

Other Supplementary Material for this manuscript includes the following:

Data S1 to S15

Supplementary Text

<u>Identification of *RTSW* plants with normal growth phenotype and application in breeding</u>

Owing to the presence of introgressed loci from wild relatives, which exhibit low sequence similarity with the corresponding regions of their cultivated relatives, recombination suppression often occurs. To obtain *RTSW* plants showing normal growth, we designed and screened a very large BC_7F_1 population [K326 × BC_6F_1 (plant 46#, with the genotype *rtsw/RTSW*, *def1/DEF1*)], comprising over 160,000 seedlings (Fig. 2D, fig. S4B, C). We initially focused on the leaf deformation phenotype and systematically removed any deformed individual identified by the naked eye. Seedlings exhibiting thickened, ribbon-shaped leaves, irregular venation, and swollen veins were classified as deformed. After five rounds of screening at the seedling stage, we obtained approximately 12,000 individuals with normal morphology for marker-assisted selection (Fig. 2D, fig. S4D).

Using the NaChr3_58.4M and NaChr3_64.6M markers, which flank the *RTSW* introgression segment in plant #46, we identified 18 recombination events among the 12,000 individuals. All remaining individuals tested negative for both markers (homozygous for the K326 segment, fig. S4E). Interestingly, all 18 recombinants tested positive for the NaChr3_58.4M marker but negative for the NaChr3_64.6M marker (fig. S5A), suggesting that the *DEF2* locus may be located closer to the NaChr3_64.6M marker than to the NaChr3_58.4M marker. All 18 plants tested negative for the markers specific for the *DEF1* locus, NaChr4_2M and NaChr4_8M (fig. S5A). Among the 18 plants, we ultimately identified five plants exhibiting HR induction upon NSm infiltration (fig. S4F, G). Through the use of additional SCAR markers, we identified five *RTSW* introgression plants with shorter segments resulting from crossovers between the markers NaChr3_60M and NaChr3_64.6M (Fig. 2E, fig. S5A). Consequently, we narrowed the *DEF2* locus down to a region between the NaChr3_62.6M and NaChr3_65.7M markers, corresponding to 62.6–65.7 Mb, while the *RTSW* locus was mapped to the 58–60 Mb region on chromosome 3 of *N. alata* (Fig. 2E).

To determine whether the shorter *RTSW* introgression segments retained the same level of resistance to TSWV as in Polalta, we tested the self-pollinated F_2 population derived from plant #12, which possessed the shortest introgression segment. Virus inoculation tests revealed that all plants carrying the *RTSW* introgression segment (positive for the NaChr3_59M marker) showed extreme resistance to TSWV, while plants lacking the *RTSW* locus (negative for NaChr3_59M) were all fully susceptible to TSWV (fig. S5B)

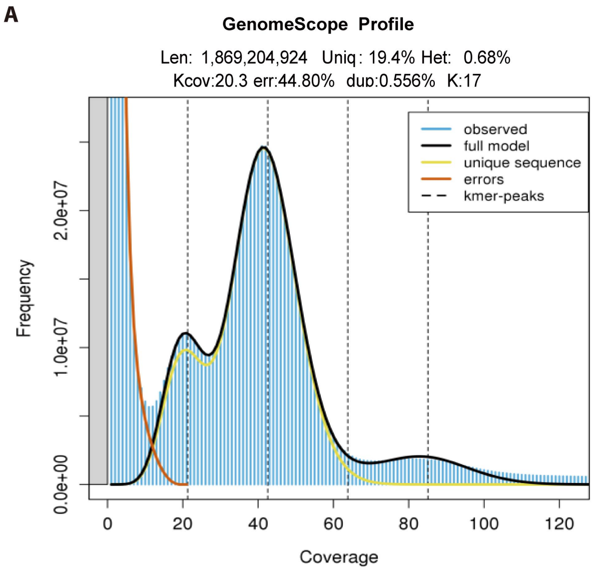

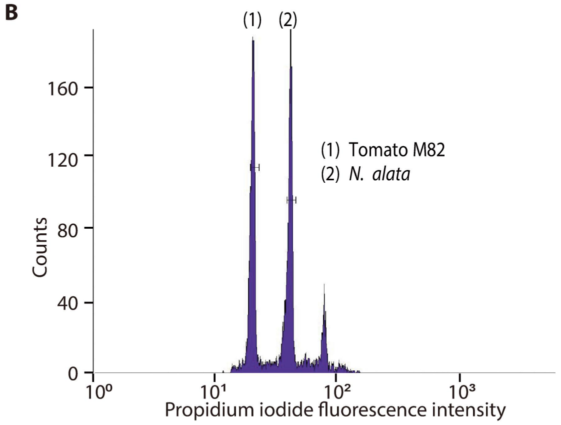

Sample	Relative fluorescence (Mean ± se)	Genome size (Gb)
Tomato M82	19.35±0.10	0.88
N. alata	38.96±0.41	1.77 ± 0.01

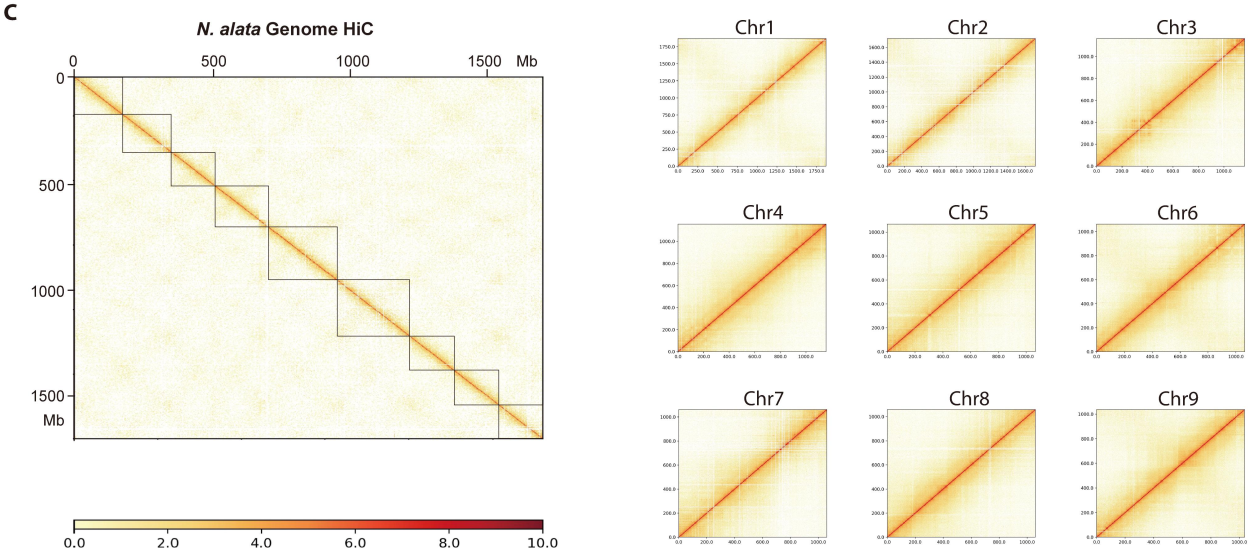

Fig. S1. **Application of *k*-mer analysis and flow cytometry to estimate the genome size of *Nicotiana alata*. (A)** *k*-mer analysis of the nuclear DNA content based on the frequency distribution of 17-mers in the sequencing reads of *N. alata*. The plot shows the GenomeScope 17-mer profile based on Illumina short-reads from the *N. alata* genome. **(B)** Flow cytometry profile of *N. alata* nuclear DNA content. Top, fluorescence intensity of propidium iodide (PI)-stained *N. alata* nuclei; bottom, estimates of genome size for *N. alata*, based on the fluorescence intensities, with *Solanum lycopersicum* 'M82' serving as an internal standard. Each sample was measured in triplicate. **(C)** Hi-C interaction matrices for the entire *N. alata* genome (left) and individual chromosomes (right). The nine *N. alata* pseudochromosomes are numbered according to their size, from largest to smallest. The interaction heatmap illustrates the normalized counts of Hi-C links within 200-kb bins, with the data presented on a logarithmic scale to highlight intrachromosomal interactions and structural organization.

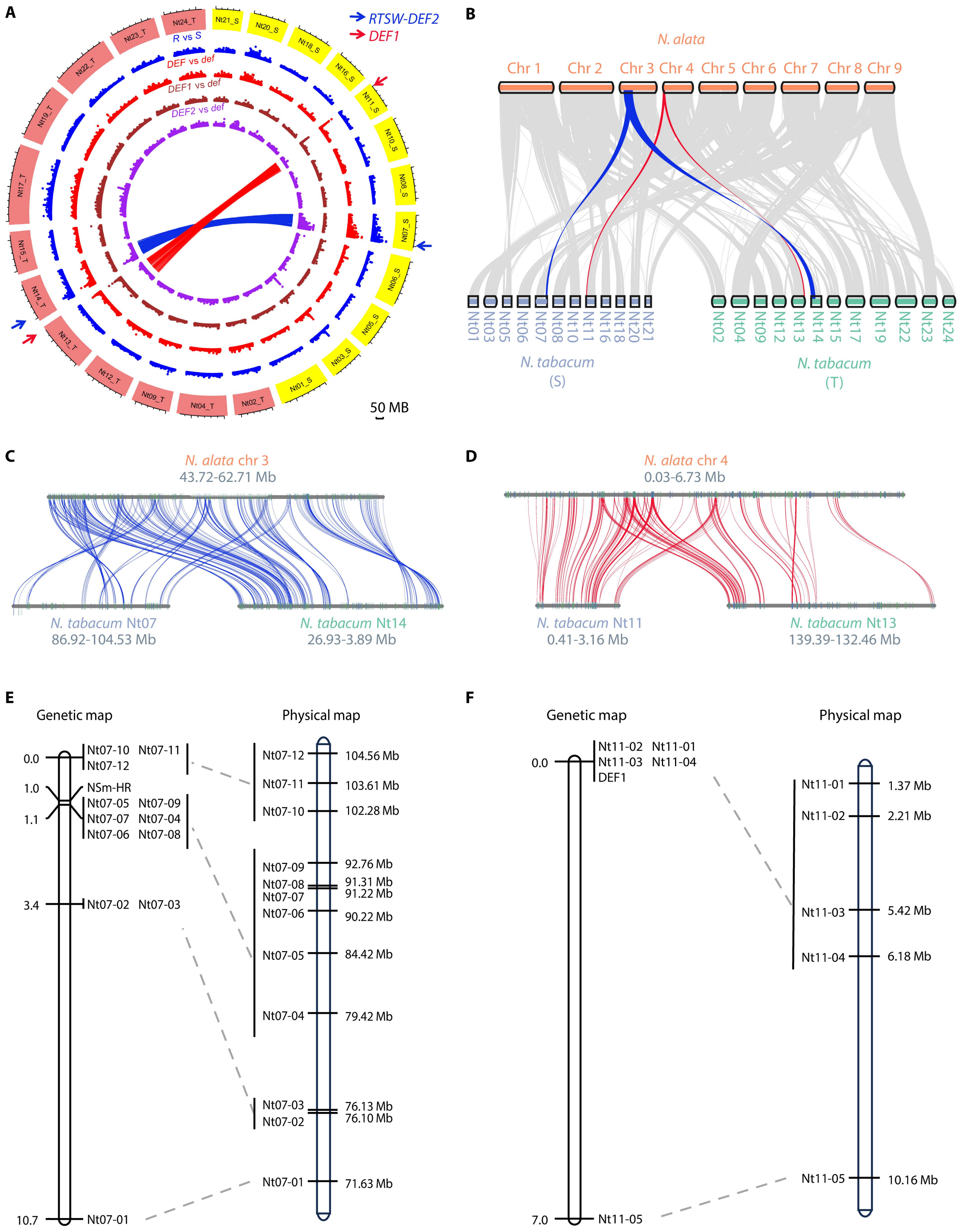

Fig. S2. Detailed mapping of the *RTSW* and *DEF* loci in K326. (A) Whole-genome sequencing bulked-segregant analysis (BSA-seq) and allele frequency difference (AFD) analysis of BC_6F_2 seedlings derived from the Polalta × K326 cross. Polalta-specific SNPs filtered from

BSA-seq mapping of the *RTSW* and *DEF* loci in a BC_6F_2 population derived from Polalta × K326 (with K326 as the recurrent parent) were mapped to the K326 genome and their frequencies plotted. Blue and red arrows indicate potential *RTSW-DEF2* and *DEF1* loci. Synteny links between target chromosomes (Nt07–Nt14 and Nt11–Nt13) are highlighted with blue and red lines, respectively. Scale bar, 50 Mb. **(B)** Macrosynteny analysis between *N. alata* and *N. tabacum*. The *RTSW-DEF2* (blue) and *DEF1* (red) loci are highlighted. **(C)** Microsynteny analysis of the *RTSW-DEF2* introgressed segment in *N. alata* and its potential homologous regions in the K326 genome. **(D)** Microsynteny analysis of the *DEF1* introgressed segment in *N. alata* and its potential homologous regions in the K326 genome. Truncated homology on Nt11 may reflect assembly gaps. **(E)** Genetic and physical maps of the *RTSW-DEF2* introgressed segments when mapped onto the K326 genome. The genetic map was developed through linkage analysis of NSm-induced hypersensitive response (NSm-HR) phenotypes in 90 BC_6F_2 lines, using their genotypes at 12 specifically developed SSR markers. The physical map of the SSR markers was determined based on the K326 genome as reference, localizing the *RTSW-DEF2* locus to Nt07 (79.42 – 104.56 Mb) using 12 SSR markers. **(F)** Genetic and physical maps of the *DEF1* locus in K326. The genetic map was constructed from linkage analysis of deformation phenotypes in 90 BC_6F_2 lines, using their genotypes across five SSR markers. Deformation phenotypes were identified through NSm-HR, with only HR-negative plants selected for genetic mapping.

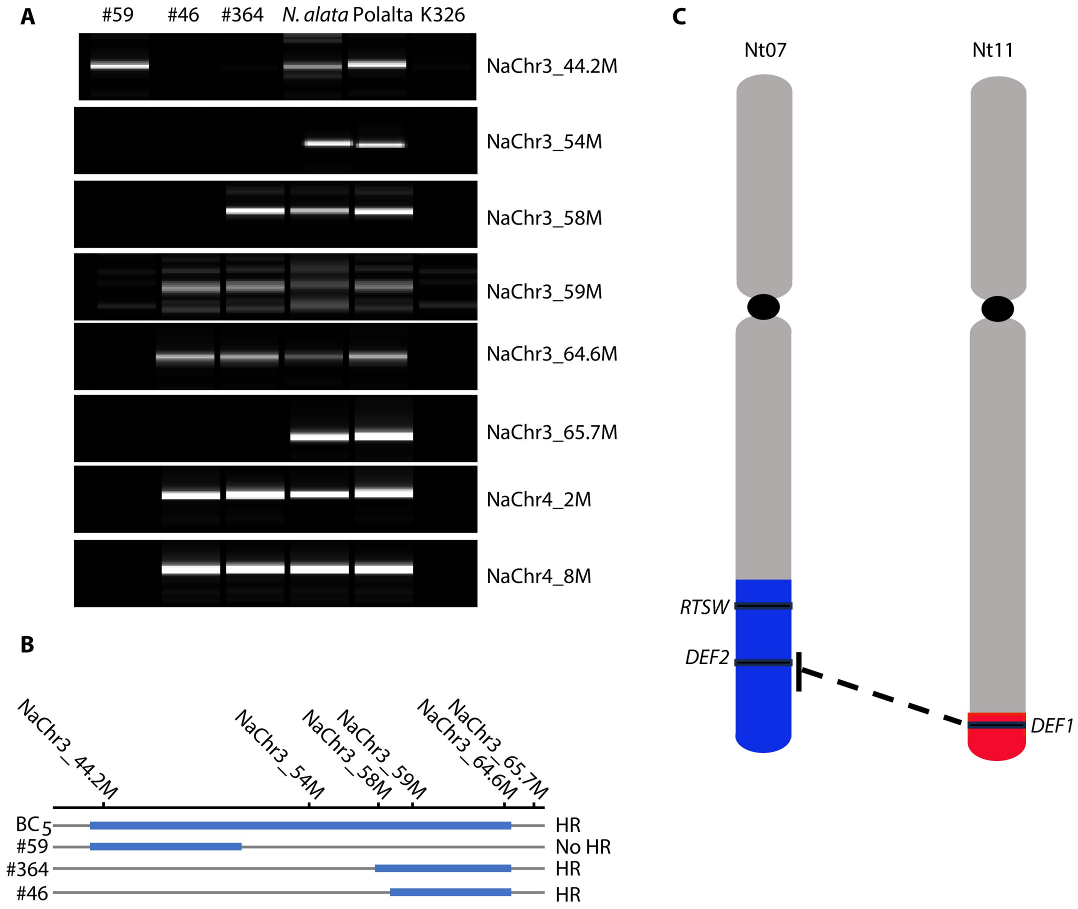

Fig. S3. Characterization and modeling of the *RTSW-DEF2* and *DEF1* introgressed segments. **(A)** PCR genotyping of three recombinants (plants #59, #46, and #364) from a BC_6F_1 population of 1,500 plants, *N. alata*, K326, and the Polalta line, using SCAR markers. SCAR markers were developed for the *RTSW-DEF2* introgressed segment spanning 44.2 Mb to 65.7 Mb on chromosome 3 and for the *DEF1* introgressed segment from 2 Mb to 8 Mb on chromosome 4 of *N. alata*. *N. alata* and Polalta served as positive controls, while K326 was the negative control. **(B)** Diagram of the *RTSW-DEF2* introgressed segments from *N. alata* in the three recombinants identified with SCAR markers shown in A. The resistance phenotype was assessed using the NSm-HR test. **(C)** Proposed model illustrating the genetic interaction between the *RTSW-DEF2* and *DEF1* loci. The gray regions represent chromosomes Nt07 and Nt11 of *N. tabacum* K326. The blue region denotes the introgressed segment from *N. alata* chromosome 3, with the predicted locations of the *RTSW* and *DEF2* genes indicated by black lines. The red region denotes the introgressed segment from *N. alata* chromosome 4, with the predicted location of the *DEF1* gene marked by a black line. The dashed line suggests a hypothetical regulatory interaction between the *DEF1* and *RTSW-DEF2* loci.

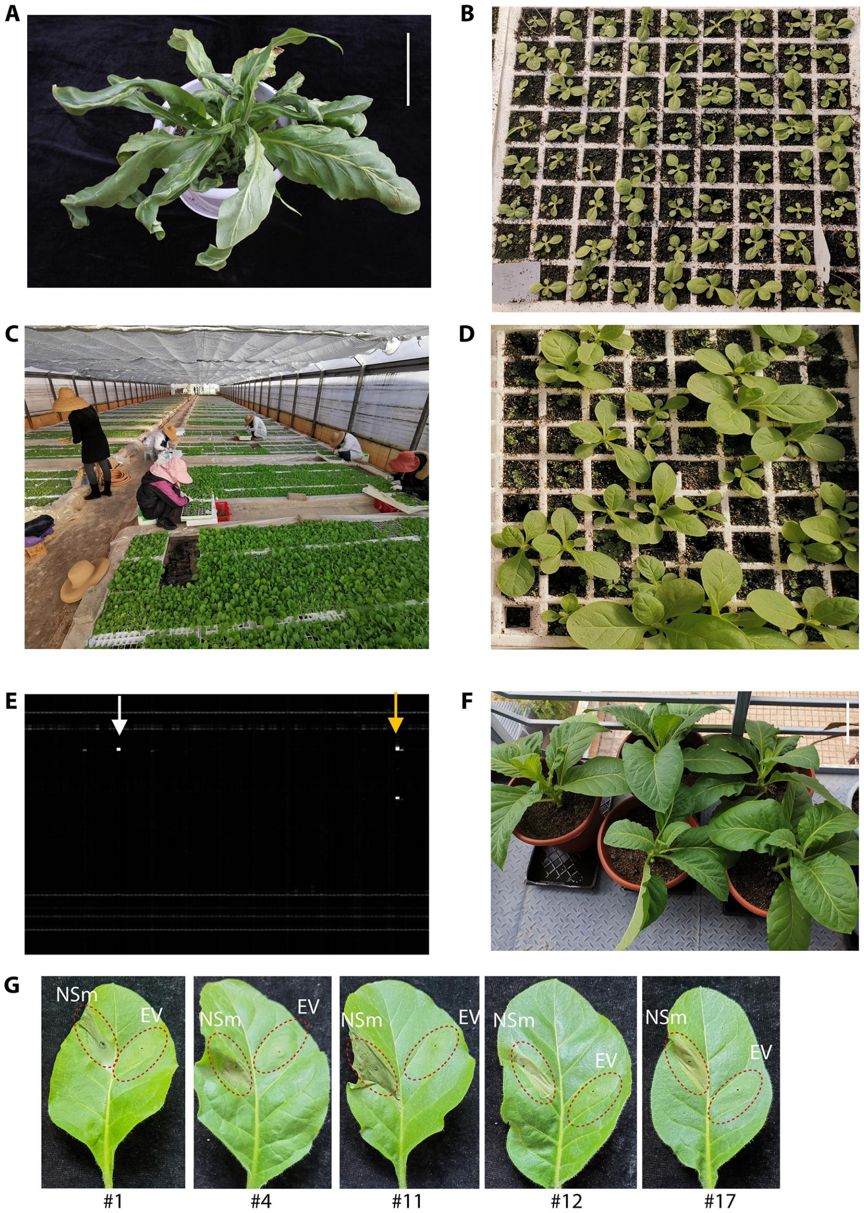

Fig. S4. **Screening for *RTSW* plants with normal growth.** **(A)** Typical morphological deformity of progeny from the Polalta line, with thickened and ribbon-shaped leaves, irregular venation, and dwarfing. Scale bar, 10 cm. **(B)** Seedling phenotypes at 30 days post sowing. All seedlings showing deformity were removed. **(C)** Manual screening by eye. More than 160,000 BC_7F_1 seedlings were grown in the greenhouse. Forty-six pools were filled with 1,104 floating trays (24 trays per pool). Around 150 seedlings were grown in each tray. **(D)** Remaining plants with normal growth after the 4th round of screening. **(E)** Molecular marker–assisted selection of the normal plants after five rounds of screening. White arrow indicates an individual with recombination in the *RTSW* locus with the bottom band missing; yellow arrow indicates Polalta with two bands as positive control. Top band indicates the NaChr3_59M marker, and bottom band indicates the NaChr3_64.6M marker. **(F)** Five resistant plants with normal growth obtained from the BC_7F_1 population. Scale bar, 10 cm. **(G)** HR test of recombinants from panel E. One

leaf from each plant was infiltrated with TSWV *NSm* (NSm, left) and empty vector (EV, right); the HR results were recorded at 3 days post inoculation (dpi).

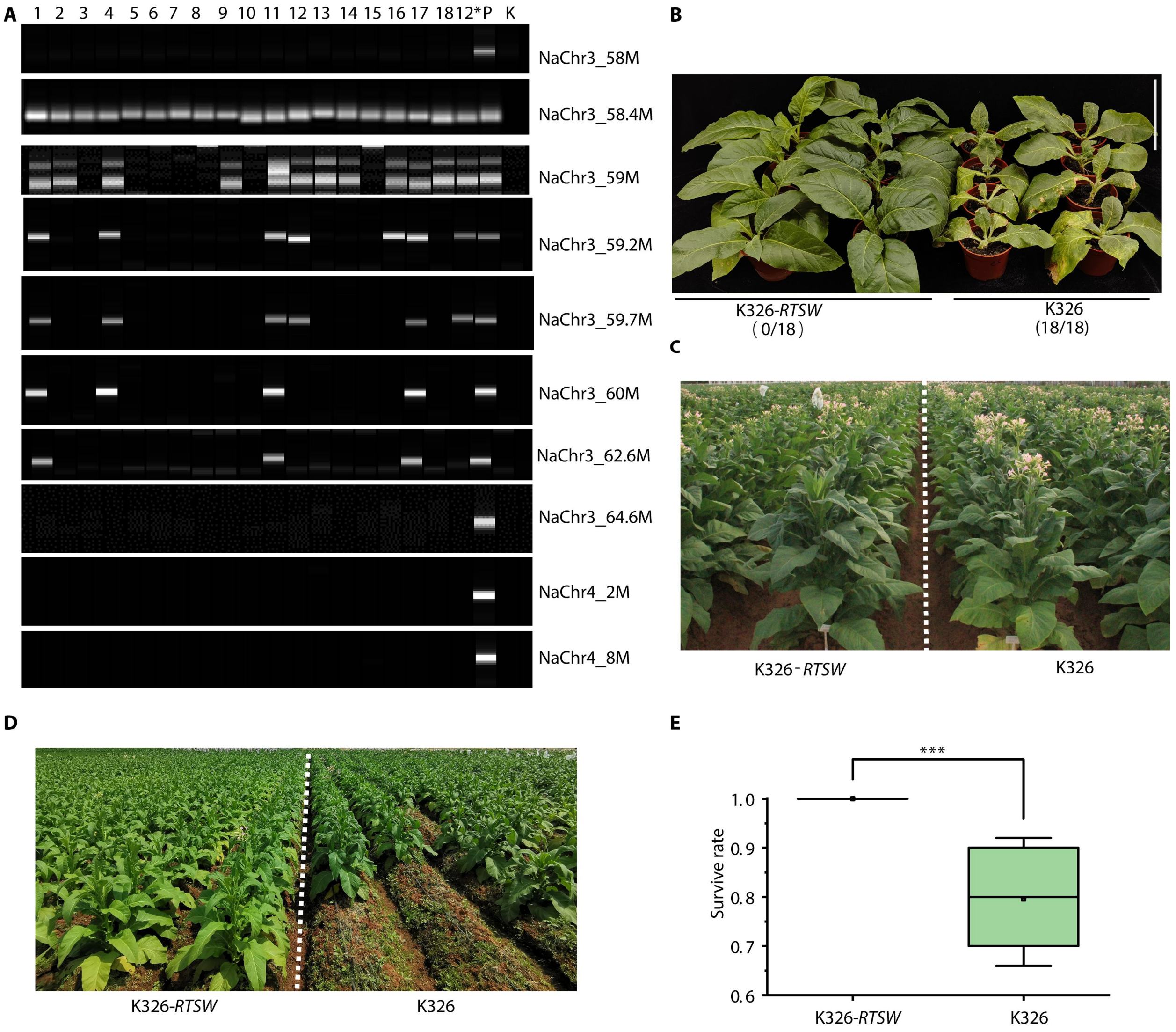

Fig. S5. Assessment of recombination events and field performance. **(A)** Marker-assisted selection of 18 plants derived from a BC_7F_1 segregating population of over 160,000 seedlings with recombination events between the tested markers. Eight contiguous SCAR markers spanning the *RTSW* introgressed segment (NaChr3_58M to NaChr3_64.6M) and two markers for the *DEF1* locus (NaChr4_2M and NaChr4_8M) were used. Each plant is designated by a number, with plant #12 shown twice for repeated testing. Polalta (P) and K326 (K) served as positive and negative controls, respectively. **(B)** In the self-pollinated F_2 progeny of plant #12, individuals carrying the *RTSW* introgressed segment (K326-*RTSW*) demonstrated immunity to TSWV, in contrast to plants lacking the *RTSW* locus (K326), which were fully susceptible at 14 dpi. The numbers indicate the ratio of plants showing typical TSWV symptoms to the total number of plants inoculated. The experiment was repeated up to three times with consistent results. Scale bar, 10 cm. **(C)** Field performance of K326-*RTSW* and K326 plants in the absence of tobacco spotted wilt disease (TSWD). The photograph was taken during the flowering stage in the field. **(D)** Field performance of K326-*RTSW* and K326 in a plot experiment with natural TSWD infection. The photograph was taken before flowering in 2024. Empty spaces in the field are areas where plants died from TSWD infection and were removed by the farmer to prevent virus

spread. **(E)** Survival rate of K326-*RTSW* and K326 plants infected with TSWD, as shown in panel D. Surviving plants in 10 randomly selected rows (47–51 plants per row) were counted, and survival rates were calculated. Boxplots show the 25th–75th percentile, with whiskers representing the full data range and center lines indicating the median. Asterisks indicate a statistically significant difference (***, $p < 0.001$) as determined by an unpaired two-sided Student's *t*-test.

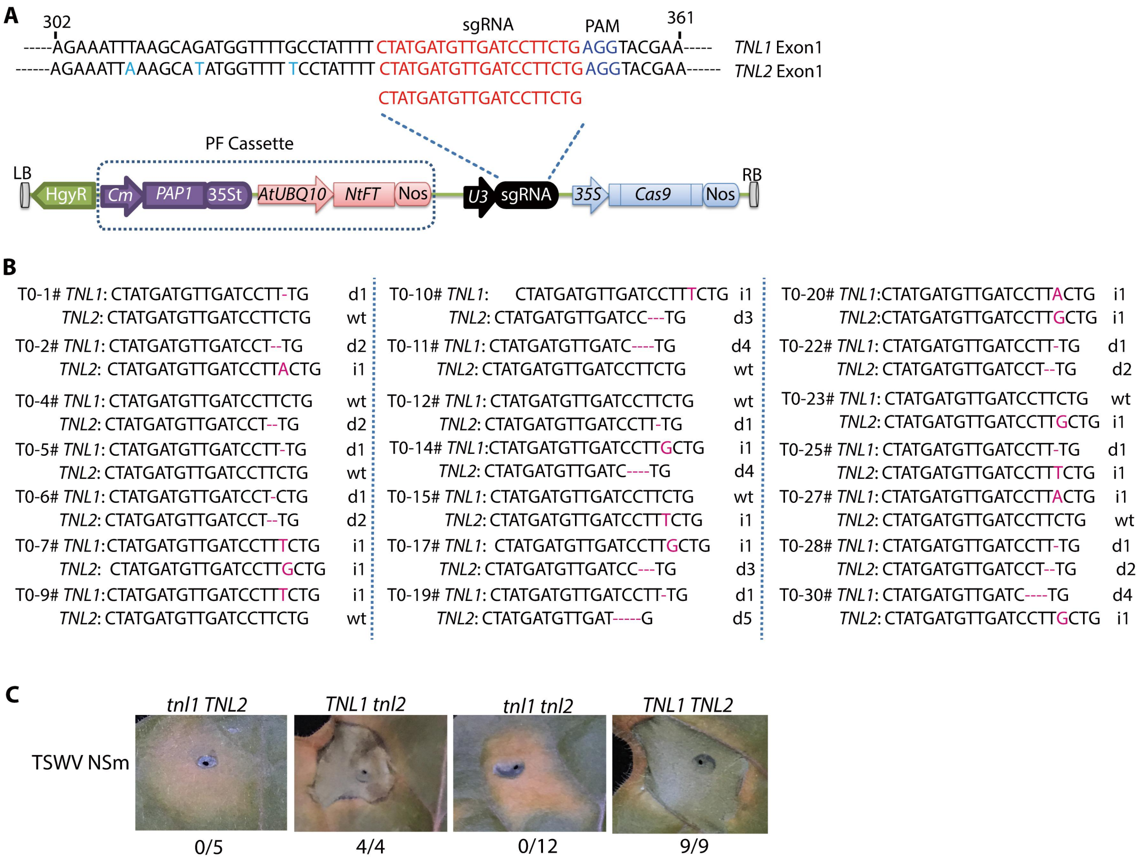

Fig. S6. Gene editing of *TNL1* and *TNL2*. (A) Diagram and partial sequence of the Cas9-PF vector armed with sgRNA, targeting the conserved region of the first exon of the *TNL1* and *TNL2* genes. The sgRNA target site is indicated in red and the protospacer adjacent motif (PAM) site is indicated in blue. **(B)** Sanger sequencing results for the detection of mutations in *TNL1* and/or *TNL2* in 21 representative gene-edited T_0 plants. Nucleotide deletions are denoted by magenta dashes, and nucleotide insertions are shown in magenta uppercase. The numbers of deleted (d#) or inserted (i#) nucleotides are shown to the right of each sequence. wt refers to the wild-type sequence without editing events. **(C)** Assessment of RTSW resistance in *TNL1*- and/or *TNL2*-edited plants by NSm-induced HR assay. Details of each editing event are presented in panel B. The numbers below each photograph represent the ratio of plants displaying HR to the total number of plants infiltrated.

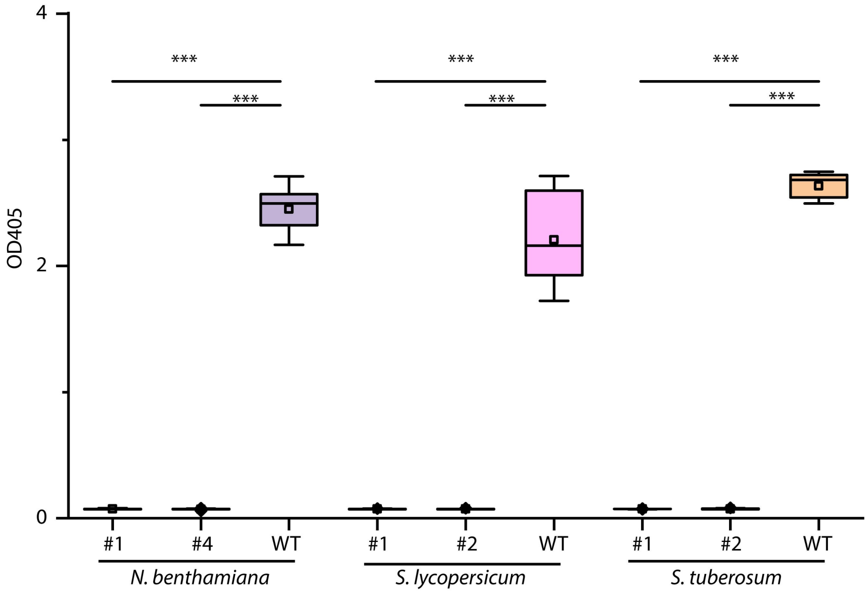

Fig. S7. Enzyme-linked immunosorbent assay (ELISA) for TSWV detection. ELISA was conducted to detect the presence of TSWV in the *RTSW* transgenic *N. benthamiana*, *S. lycopersicum*, and *S. tuberosum* plants shown in Fig. 3E. Plants inoculated with TSWV were harvested at 14 dpi, and total protein extracts were used for ELISA analysis with TSWV-specific antibodies. The optical density at 405 nm (OD_{405}) was measured from three independent experiments, 30 min after the addition of the substrate at room temperature. A minimum of six plants per line and wild-type (WT) control were assessed. Boxplots represent the 25th–75th percentiles, with whiskers showing the full range of data and center lines indicating the median. Asterisks denote a statistically significant difference (***, $p < 0.001$), as determined using unpaired two-sided Student's *t*-tests.

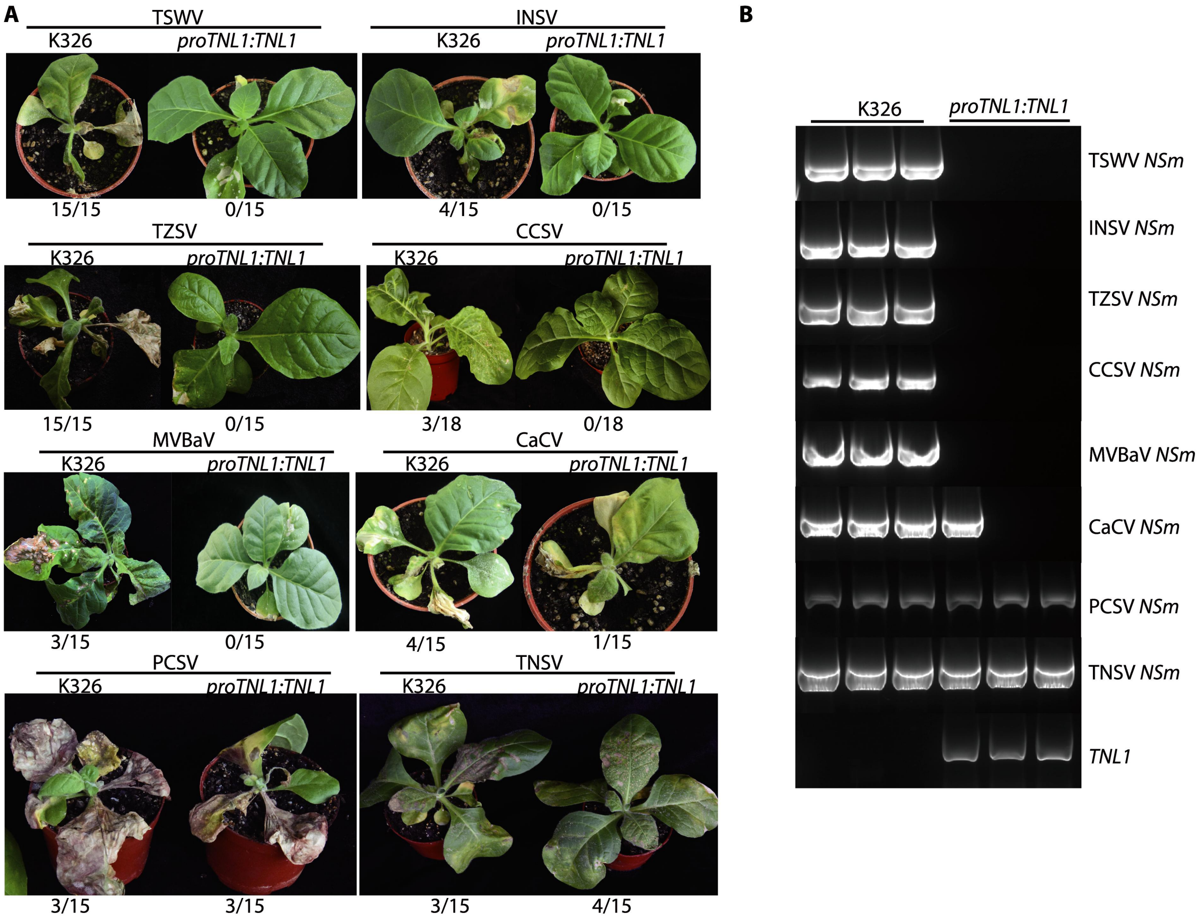

Fig. S8. Broad-spectrum resistance of *RTSW* transgenic plants. **(A)** Symptoms of wild-type (K326) and *RTSW* transgenic (*proTNL1*:*TNL1*) K326 plants inoculated with two American-type orthotospoviruses (TSWV and impatiens necrotic spot orthotospovirus [INSV]) and six Eurasian-type orthotospoviruses (tomato zonate spot orthotospovirus [TZSV], tomato necrotic spot orthotospovirus [TNSV], pepper chlorotic spot orthotospovirus [PCSV], calla lily chlorotic spot orthotospovirus [CCSV], mulberry vein banding associated orthotospovirus [MVBaV], and capsicum chlorosis orthotospovirus [CaCV]). The photographs were taken at 14 dpi. The numbers in each image indicate the ratio of plants displaying typical symptoms of orthotospoviruses to the total number of inoculated plants. **(B)** PCR validation of virus proliferation in the plants shown in (A). At least three plants from each treatment group were selected. Total RNA and DNA were extracted for virus testing and *RTSW* genotyping. Specific primers for the different *NSm* genes were used to detect the presence of the orthotospovirus RNAs.

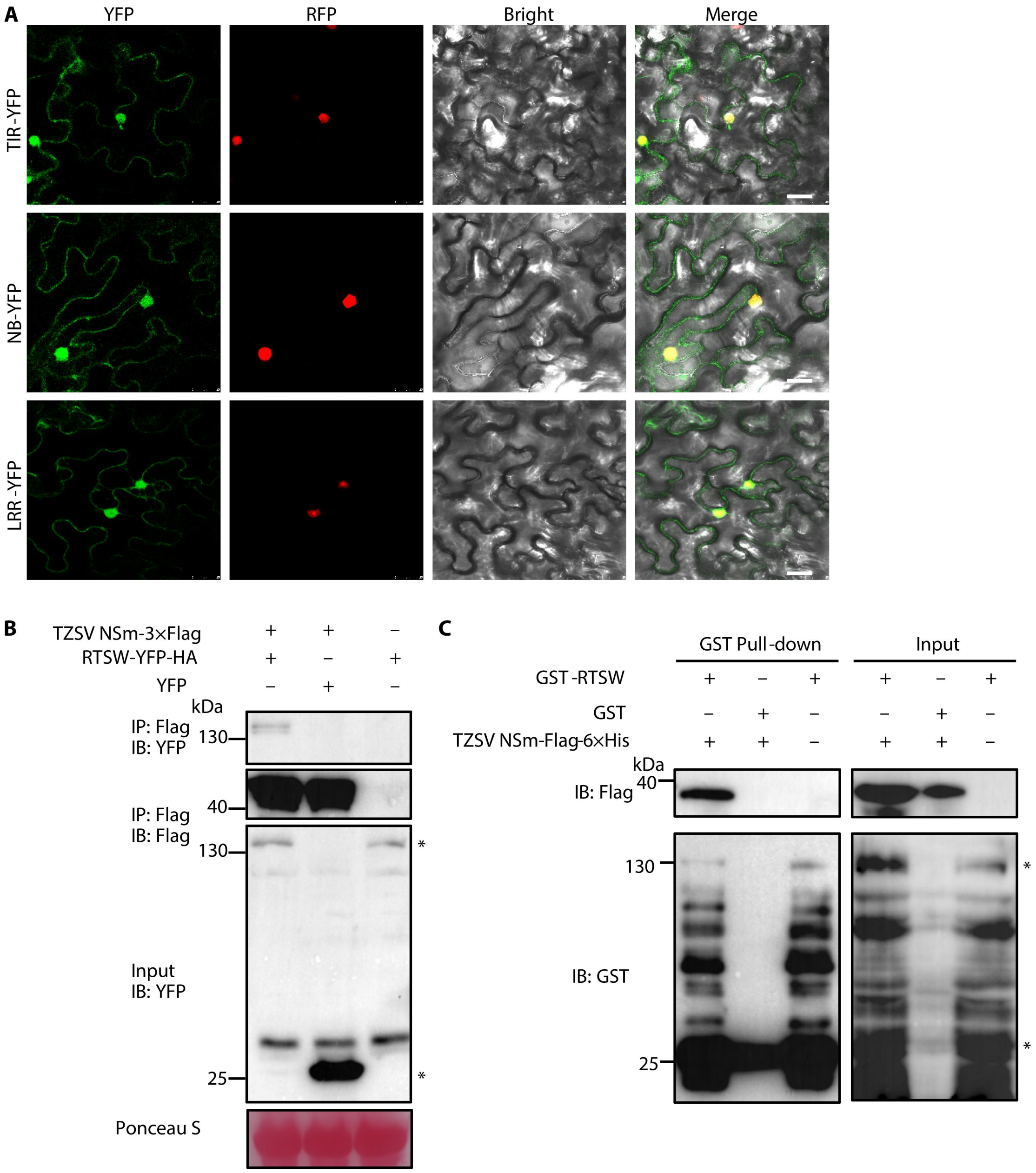

Fig. S9. Subcellular localization and interaction analysis of RTSW. **(A)** Subcellular localization of the individual TIR, NB, and LRR domains of RTSW. These domains were fused to YFP and their encoding constructs were individually co-infiltrated into *N. benthamiana* leaves with *H2B-RFP* as a nuclear marker. Confocal microscopy imaging was performed at 24 hours post infiltration (hpi). YFP and RFP signals are depicted in green and red, respectively. Scale bars, 25 µm. **(B)** Co-immunoprecipitation (Co-IP) assay to investigate the interaction between RTSW and TZSV NSm. TZSV NSm-3×Flag was immunoprecipitated with anti-FLAG antibodies from total protein extracted from *N. benthamiana* leaves co-infiltrated with *TZSV NSm-3×Flag* and *RTSW-YFP-HA*; proteins were detected using anti-FLAG or anti-YFP antibodies. The asterisk denotes the specific band with the predicted size for RTSW-YFP-HA (top) or YFP (bottom). **(C)** *In vitro* GST pull-down assay to examine the interaction between RTSW and TZSV NSm. GST alone, GST-RTSW, and TZSV NSm tagged with Flag-6xHis

(TZSV NSm-Flag-6×His) were produced and purified from *Escherichia coli*. The supernatant containing TZSV NSm-Flag-6×His was incubated with GST-RTSW or GST and subsequently pulled down with glutathione-Sepharose beads. Immunoblots were probed with anti-GST or anti-Flag antibodies. The asterisks indicate the specific band with the predicted size for the GST fusion (top) or GST alone (bottom).

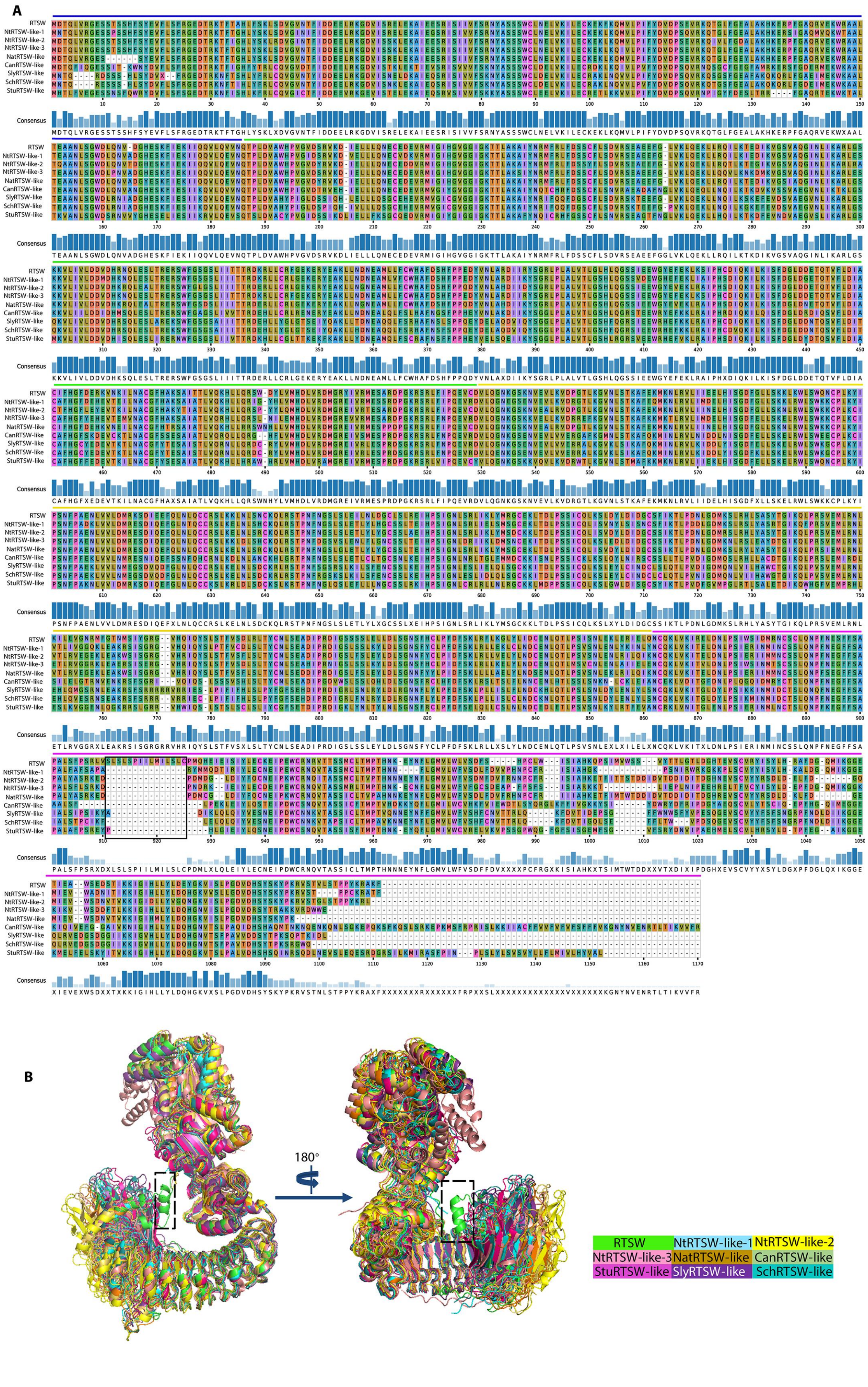
A
RTSW
NtRTSW-like-1
NtRTSW-like-2
NtRTSW-like-3
NatRTSW-like
CanRTSW-like
SlyRTSW-like
SchRTSW-like
StuRTSW-like
Consensus
B
180°
RTSW
NtRTSW-like-1
NtRTSW-like-2
NtRTSW-like-3
NatRTSW-like
CanRTSW-like
StuRTSW-like
SlyRTSW-like
SchRTSW-like

Fig. S10. Comparison of RTSW and its homologs. **(A)** Full-length protein sequence alignment of RTSW and its homologs using ClustalW within MEGA X. The TIR, NB, LRR, and C-JID domains are highlighted by blue, green, yellow, and purple lines, respectively, above the aligned sequences. A distinctive 15-amino-acid motif in RTSW, spanning residues 905–919, is indicated in a black box. **(B)** Protein structure overlay of RTSW and its homologs as presented in A, with each protein shown in a different color for clarity. The specific alpha helix of RTSW is indicated by the black dashed rectangle. The homologs include three RTSW-like proteins from *N. tabacum*, NtRTSW-like-1 (NCBI Accession No: XP_016472620.1, AlphaFold ID: A0A1S4A7L0_A), NtRTSW-like-2 (NCBI Accession No: XP_016457588.1, AlphaFold ID: A0A1S3YZP1_A), and NtRTSW-like-3 (NCBI Accession No: XP_016507158.1, AlphaFold ID: A0A1S4D1G2_A); one from *N. attenuata*, NattRTSW-like (NCBI Accession No: XP_019265600.1); one from *S. lycopersicum*, SlyRTSW-like (NCBI Accession No: XP_025888731.1); one from *S. chinense*, SchRTSW-like (NCBI Accession No: TMW88998.1, AlphaFold ID: A0A6N2B4R0_A); one from *Capsicum annuum*, CanRTSW-like (NCBI Accession No: XP_016566173.2, AlphaFold ID: A0A1U8GBU4_A); and one from *S. tuberosum*, StuRTSW-like (NCBI Accession No: XP_015170895.1).

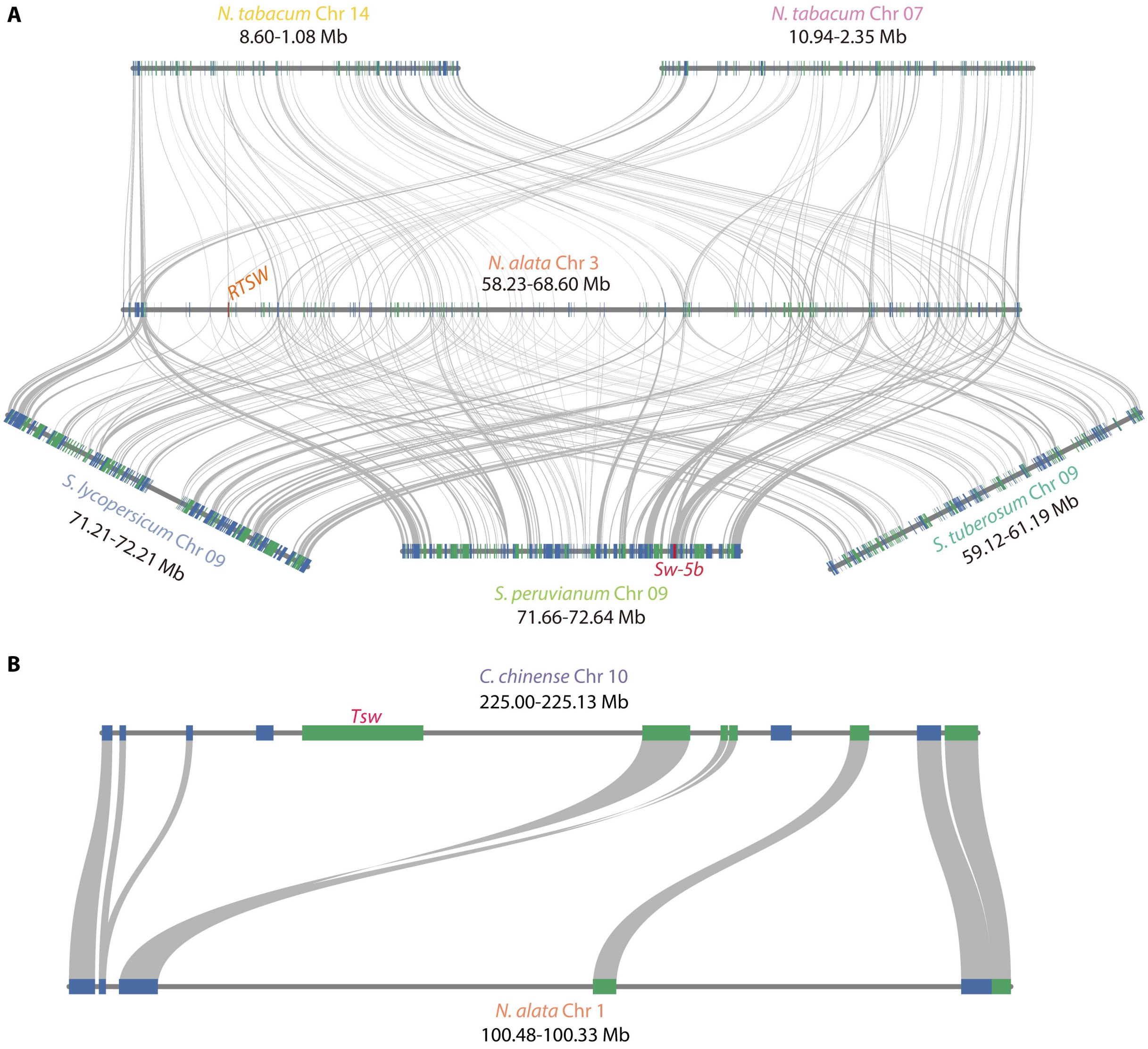

Fig. S11. Microsynteny of the *RTSW*, *Sw-5b*, and *Tsw* genomic regions. **(A)** Detailed microsynteny analysis of the genetic regions containing the *RTSW* allele on *N. alata* chromosome 3 (Chr 3) and the *Sw-5b* allele on *S. peruvianum* chromosome 9 (Chr 09), as well as other Solanaceae species. The diagram is presented in Fig. 5C. The *RTSW* gene is depicted in dark yellow, and the *Sw-5b* gene in purple. To provide a more precise representation of the genetic landscape, a recently updated allotetraploid *N. tabacum* genome reference was used for this analysis. Specifically, chromosome 7 (Chr 07) and chromosome 14 (Chr 14) from the S and T subgenomes were selected for the microsynteny assessment. **(B)** Microsynteny analysis of the genetic regions containing the *Tsw* allele on chromosome 10 (Chr 10) of *C. chinense* and *N. alata* chromosome 1 (Chr 1). The regions selected for analysis are indicated with their respective positions on each chromosome. Genes on the positive and negative strands are depicted as solid green and blue bars, respectively.

Legends for data S1-S15 (separate file) and data S16 (see next page).

Data S1. *Nicotiana alata* sequencing strategy description and HiC Sequencing data
Data S2. Scaffolds statistics summary
Data S3. Summary of the final genome assembly
Data S4. Chromosome Sorting
Data S5. BUSCO assessment of genome assembly for *N. alata*
Data S6. Predicted repetitive elements in *N. alata* genome
Data S7. The prediction of gene structures of *N. alata*
Data S8. Genome annotation completeness
Data S9. Functional annotation in *N. alata* genome
Data S10. Annotation of Non-coding RNA
Data S11. NLR gene numbers in selected Solanaceae Species
Data S12. Segregation ratio of resistance and deformity traits in BC_6F_2 population (Chi-square test)
Data S13. HR and genotyping test of all the deformed individuals without *DEF1* (*DEF2/def1*)
Data S14. Annotated genes in the 59.2-60.0 Mb region of *N. alata* Chromsome 3
Data S15. Primers used in this study
Data S16.Uncropped immunoblots corresponding to Fig. 4D

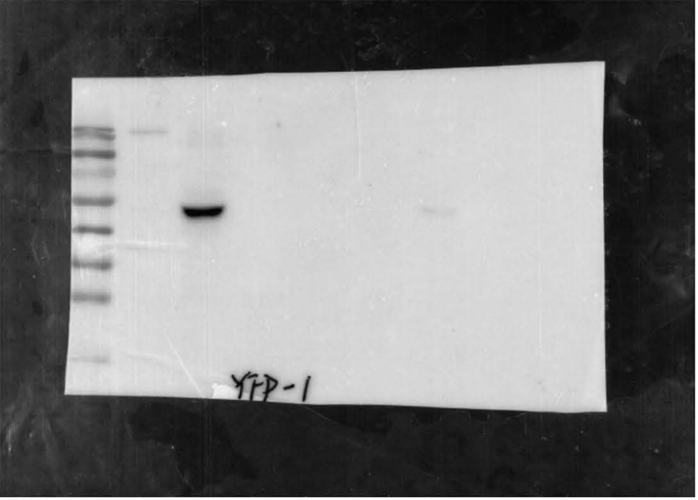

IP: Flag
IB: YFP

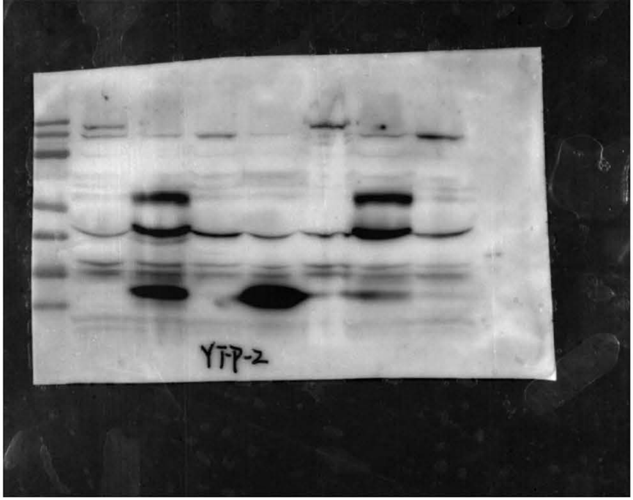

Input
IB: YFP

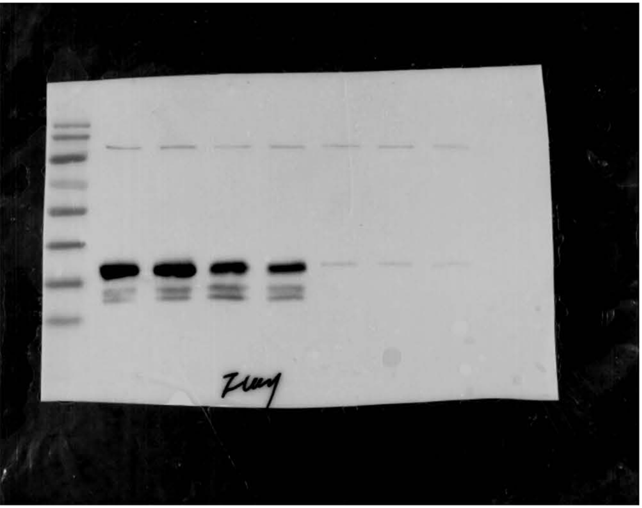

IP: Flag
IB: Flag

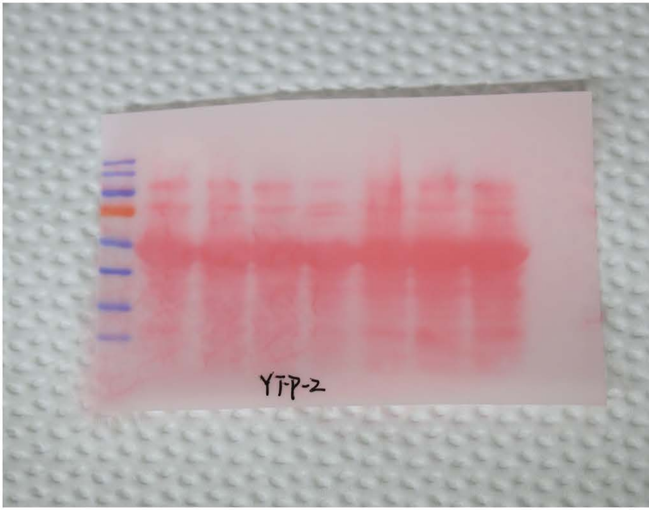

Ponseau S
